# The Impact of Web-Based Physical Activity Interventions on Depression and Anxiety Among College Students: Randomized Experimental Trial

**DOI:** 10.2196/31839

**Published:** 2022-04-01

**Authors:** Andy Murray, Michele Marenus, Ana Cahuas, Kathryn Friedman, Haley Ottensoser, Varun Kumaravel, Julia Sanowski, Weiyun Chen

**Affiliations:** 1 Physical Activity and Health Laboratory School of Kinesiology University of Michigan Ann Arbor, MI United States; 2 Department of Psychology School of Literature, Science, and the Arts University of Michigan Ann Arbor, MI United States

**Keywords:** depression, anxiety, college students, mindfulness, aerobic exercise, resistance training, web-based intervention

## Abstract

**Background:**

Depression and anxiety are growing issues for college students, with both aerobic resistance training and mindfulness yoga exercises known to be effective in reducing symptoms and severity. However, no known research is available comparing these 2 depression and anxiety interventions simultaneously and in a web-based environment.

**Objective:**

This study aims to determine the effects of a web-based aerobic resistance exercise intervention (WeActive) and a web-based yoga mindfulness exercise intervention (WeMindful) on depression and anxiety symptoms in college students.

**Methods:**

The participants were 77 college students who anonymously completed a Qualtrics survey, including the Generalized Anxiety Disorder Scale and the Major Depression Inventory at baseline and after the intervention. Participants were randomly assigned to either the WeActive or WeMindful group and underwent two 30-minute web-based aerobic resistance exercise lessons or yoga mindfulness lessons per week for 8 weeks.

**Results:**

The results of analysis of covariance with repeated measures indicated that although not statistically significant, both groups showed a notable decrease in anxiety with a marginally significant main effect of time (*F*_1_=3.485; *P*=.07; *η*^2^=0.047) but no significant main effect of group and no significant interaction effect of time with group. The 2 intervention groups experienced a significant decrease in depression with the main effect of time (*F*=3.892; *P*=.05; *η*^2^=0.052). There was no significant main effect of group or interaction effect of time with group for depression.

**Conclusions:**

College students in both WeActive and WeMindful groups experienced a significant decrease in depression symptoms and a decrease, although not significant, in anxiety as well. The study suggests that web-based WeActive and WeMindful interventions are effective approaches to managing US college students’ depression and anxiety during a pandemic.

## Introduction

### Background

It has been well-documented that mental health issues are increasing within a college student population [1,2]. This increase in mental health problems, such as anxiety and depression, seems to have been exacerbated by the onset of the COVID-19 pandemic [3-6], making the task of determining effective reduction strategies more pressing. Examining for potential increases in depression and anxiety issues due to the COVID-19 pandemic, global research has found that mental health problems have increased both in prevalence and severity in several countries. In China, 31% and 41.8% of 1396 surveyed college students experienced depression and anxiety, respectively [5]. Corroborating these findings, a separate longitudinal study on 66 Chinese college students found that qualities of mental health, including anxiety, were negatively affected by the pandemic [6]. In Italy, research has found that 27.8% of 400 analyzed college students and employees displayed notable depressive symptoms and 34.3% displayed notable anxiety symptoms [3]. In the United States, 44% of 195 interviewed college students reported an increase in depressive symptoms, whereas 71% of the same sample of students reported an increase in stress and anxiety symptoms [4]. In a meta-analysis examining the percentage increases in self-reported anxiety and depression worldwide, researchers found an increase in anxiety from 19% to 37% and an increase in depression from 21% to 54% after March 1, 2020, the time at which the SARS-CoV-2 virus started to rapidly spread worldwide [7]. Many colleges and mental health researchers have focused on finding ways to prevent or treat this rising rate of anxiety and depression [4,8,9], as these mental health problems are thought to have been aggravated by the onset of the global COVID-19 pandemic. Recently, attention has been given to potential lifestyle-based anxiety and depression prevention and treatment strategies, such as through the use of physical activity and mindfulness exercise interventions.

The 2018 Physical Activity Guidelines for Americans recommends that adults participate in 150 minutes of moderate-intensity aerobic activity per week or 75 minutes of vigorous-intensity aerobic activity per week or a combination of the 2 intensities [10]. Research has shown that engaging in physical activity, even in amounts that are less than those recommended by the aforementioned guidelines, can lessen the severity of mental health disorder symptoms, including anxiety and depression [5,11-15]. Depression, in particular, seems to experience the greatest benefits from increased physical activity time, including through group exercises and sports play [16] and through outdoor activities [17]. Anxiety has also been shown to decrease with participation in either individual or group sport–based exercises, with a higher frequency of physical activity correlated with lower levels of both anxiety and depression [13].

Web-based physical activity interventions have also been shown to be associated with reductions in depression and anxiety symptoms in a collegiate population [18]. Recent research on physical activity and mental health has found that more than half of all college students have not participated in an adequate amount of physical activity during the COVID-19 pandemic [5]. Researchers have discovered that individuals who have participated in high-volume and high-frequency structured exercise during the pandemic showed a decrease in depression and anxiety compared with inactive individuals [19]. At the time of study completion, the COVID-19 pandemic had not yet ended, and the lasting effects of this lack of activity in most college students were not yet known. However, as physical activity has been established to have a clear negative correlation with anxiety and depression symptoms, it can be reasonably predicted that inactive students may have more severe long-term mental health effects in the near future.

College students’ mental health has also been found to improve with the practice of mindfulness activities, which are designed to allow participants to practice self-awareness of sensation and feeling [12,20-22]. In particular, mindfulness practices of meditation [21-23] and awareness and breathing exercises [21] have been shown to be correlated with improvements in college students’ anxiety and depression issues. Yoga exercises, which combine aspects of mindfulness practices and physical activity, have also been shown to be effective in reducing depression, anxiety, and overall stress [21,23]. In addition, research has found a connection between mindfulness exercise practice and other positive psychological constructs, such as psychological flexibility [24] and nonreactivity [25], which, in turn, further decreases mental distress. Similarly, practicing mindfulness has been shown to mediate negative psychological factors, such as intolerance of uncertainty [26], and reduce anxiety symptoms that occur in connection to these negative factors.

In a web-based setting, the use of mindfulness-based interventions to reduce anxiety and depression symptoms in college students has demonstrated similar results to those of in-person interventions [27,28]. An experimental trial with 72 participants on mobile game–based meditation found a significant decrease in depression scores in the intervention group [28]. An 8-week web-based mindfulness study by Ahmad et al [27] with 113 student participants compared a full-time mindfulness virtual community (MVC) group, a part-time MVC group, a cognitive behavioral therapy group, and a waitlist control group. The study by Ahmad et al [27] found statistically significant reductions in depression scores in the full- and part-time MVC groups as well as statistically significant reductions in anxiety scores for the part-time MVC group [27]. However, not all studies on mindfulness-promotion interventions have produced significant positive changes in anxiety and depression. For example, a study examined the changes in perceived stress, anxiety, and depression in students over the course of a semester and found no statistically significant differences among the control group, mindfulness and yoga group, and stress management class group [29]. Similarly, a study examining college students’ anxiety and depression after using a mindfulness smartphone app for 5 weeks did not find significant changes from the baseline test to the postintervention test [30]. Despite the few studies that have not shown a correlation between mental health problems and mindfulness practices, most studies examining this topic have found a positive connection [12,20-28].

Overall, there is clear and repeated evidence showing that practicing mindfulness activities or physical activities can positively affect mental health problems. One study compared the influence of mindfulness exercises with that of physical activity on stress, anxiety, and depression in the adult population and found significant reductions in the severity of mental health symptoms across both interventions [31]. Another study examined the impact of physical education activities and mindfulness activities on reducing the severity of anxiety and depression in 125 university students [32]. The results indicated significant reductions in these variables in the mindfulness group, with nonsignificant but observable reductions in the physical activity group, suggesting that mindfulness practices may be more effective in combating anxiety and depression symptoms [32]. In contrast, a recent systematic review comparing the mental health effects of mindfulness-based interventions with exercise interventions in collegiate populations found that the effect size on depression and anxiety was greater with exercise interventions than with mindfulness interventions [12]. To date, few studies have examined the influence of the web-based application of these 2 intervention strategies. Furthermore, there are no intervention studies that have directly compared mindfulness exercises with physical activity using a web-based format during the pandemic. In addition, with contradictory information on intervention effects on reducing mental health problems, there is a critical need to explore the effects of web-based or Zoom-based physical activity and mindfulness exercise in counteracting mental illness symptoms. In addition, the impact of the COVID-19 pandemic on the average mental health status of college students may have complicated the ways in which mindfulness and physical activity interventions interact with anxiety and depression symptoms, furthering the need to understand the utility of these interventions. To the best of our knowledge, there is a lack of research on the effects of web-based aerobic and resistance exercises or yoga with mindfulness on college student depression and anxiety in a midpandemic environment.

### Purpose and Hypothesis

Therefore, this study aims to compare the effects of a web-based aerobic resistance exercise intervention (WeActive) and a web-based yoga mindfulness intervention (WeMindful) on depression and anxiety in college students during the winter semester of 2021. It was hypothesized that both intervention groups would produce improvements in depression and anxiety but that the fitness intervention would show slightly higher positive improvements in both measures. As fitness and mindful exercise interventions have been shown to assist with depression and anxiety improvement, the significance of this study is in determining the effectiveness of web-based fitness exercise and mindfulness exercise interventions on improving student mental health in the midst of a pandemic.

## Methods

### Participants and Study Design

**Figure 1:**
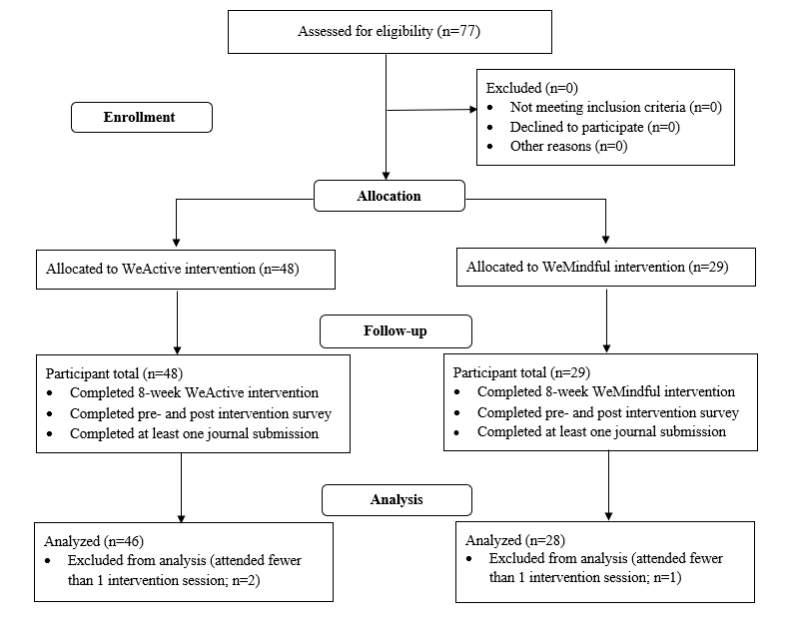
CONSORT (Consolidated Standards of Reporting Trials) recruitment and study design diagram.

We recruited college students from a large public university in the Midwestern region of the United States to participate in a randomized quasi-experimental study during the spring semester of 2021. We used several recruiting strategies, including the targeted email response system, the university’s canvas learning management platform, undergraduate and graduate bulletins, and university social media pages. The eligibility criteria included being at least 18 years of age, having current status as a student at the university, and having consistent access to the internet and to the web-based conferencing app Zoom (Zoom Video Communications). Participant enrollment information is available in Figure 1.

As seen in Figure 1, after the pre-evaluation, 77 participants attended the 8-week intervention components and completed the postintervention test. We calculated the study sample size with an effect size, Cohen *d*=0.80, on college students’ anxiety [33], 2-tailed with an α level of .05 and a power of 0.80 using G*Power 3.1.9.7 software. The results showed that the total required sample size of the study was 52, with 26 students in each intervention group. On the basis of our previous study’s adherence rate of >90% and an estimated program dropout rate of 10%, we needed to recruit a targeted sample size of 57 college students. Our sample size of 77 participants exceeded the required sample size of 57.

### Intervention Conditions

#### Overview

Both intervention groups were asked to attend two 30-minute group exercise classes via Zoom twice per week. Participants opted to attend 1 synchronized Zoom exercise lesson and 1 asynchronized lesson or 2 asynchronized lessons that were Zoom-recorded and uploaded to the University Canvas study-related website. The asynchronous recording contained the same lesson content from the synchronous live session, meaning content was repeated for the second session each week. In addition, all participants were asked to attend synchronous, researcher-led peer coaching sessions that took place once every 2 weeks for 30 minutes.

The intervention lasted for 8 weeks over the course of the winter 2021 semester. Before the intervention started, participants were given 1 week to complete a preintervention Qualtrics survey that included anxiety and depression measures. Immediately after the intervention period, the participants were again given 1 week to complete a postintervention Qualtrics survey that included the same anxiety and depression measures used in the preintervention survey.

#### WeActive Intervention

The objectives of the WeActive intervention were to help the participants engage in at least 150 minutes of moderate to vigorous physical activity per week and improve their abilities to achieve the recommended amount of weekly moderate to vigorous physical activity minutes throughout the intervention. The participants were instructed to attend two 30-minute exercise classes via Zoom per week for 8 weeks. The class duration was set to 30 minutes to maximize participant retention while still allowing participants to achieve an amount of exercise that has been shown to be beneficial [17]. The lessons were taught by a student instructor who had previously taken the University of Michigan classes *Methods of Instruction for Exercises* and *Fundamentals of Strength and Conditioning*. In addition, the WeActive student instructor was a certified strength and conditioning specialist and a graduate student with ≥5 years of experience in personal training. Each exercise lesson used a structured format, consisting of 5 minutes of warm-up (eg, marching in place and dynamic stretching), 20 minutes of both aerobic and resistance exercises (eg, squats and pushups and high knees and jumps), and 5 minutes of cooldown with static stretching exercises (eg, hamstring and quadriceps static stretching when lower body exercises were used in the session). For the first 4 weeks of the intervention, the lesson focused on introducing participants to low to moderate impact resistance and cardio exercises. As lessons continued, exercise difficulty progressed (eg, from a 2-leg bodyweight squat to a moving lunge). The last 4 weeks of the intervention included high-intensity exercise with a greater cardiovascular focus.

#### WeMindful Intervention

The WeMindful group was instructed to participate in two 30-minute web-based yoga mindfulness classes per week for 8 weeks. The use of yoga, which incorporates movement with breathing and self-awareness, as a primary mindfulness exercise has been supported by previous research [21,23]. The lessons were taught by a student instructor who had previously taken the University of Michigan classes *Methods of Instruction for Exercises* and *Fundamentals of Strength and Conditioning*. In addition, the WeMindful student instructor was a junior undergraduate student studying movement science with 3 years of experience teaching yoga and dance classes. Class content consisted of a 5- to 7-minute–long movement and breathing-based warm-up (eg, focusing on space and deep breathing), followed by 15 to 20 minutes of learning and practicing sequences of basic yoga poses (eg, cat-cow and triangle pose) and ending with a 3 to 5 minutes of mindfulness relaxation (eg, reading meditation scripts). Yoga poses used in the classes often included accommodations for more difficult positions if participants needed to decrease difficulty. The lessons progressed by adding 1-3 new yoga poses per week for the first 3 weeks of lessons, followed by the fourth week that consisted of an overview of all poses learned up to that point. The fifth through eighth weeks followed a similar format, with weeks 5, 6, and 7 consisting of new poses that were reviewed during week 8.

### Intervention Implementation Strategies

To facilitate the participants to attend the WeActive or WeMindful exercise lessons and to provide ongoing emotional, informational, and appraisal support for them, we offered all participants in both groups with four 30-minute peer coaching sessions conducted via Zoom, with 1 session every 2 weeks. Each peer coaching session included experience and feedback sharing from participants, barrier or challenge reflections, and participant suggestions for class content. The focus of the first peer coaching session was on orienting and introducing participants to the session formatting, which included goal-setting prompts, self-regulation prompts, self-monitoring reflection, and time for social support. The second, third, and fourth peer coaching sessions delved deeper into obtaining participant feedback on any challenges or preferences they had during the main intervention group components, along with continued goal-setting, self-regulation, and self-monitoring exercises. The peer coaching sessions were led by 1 doctoral student and 1 other undergraduate student of the research team. In addition to peer coaching sessions, all participants were instructed to complete weekly journals that asked participants to provide their weekly attendance for both synchronous and asynchronous classes. In addition, participants received encouraging messages on the web from the research team every Thursday, with content including praise and motivational phrasing.

### Intervention Outcome Measures

#### Anxiety Measure

This study used the 7-item Generalized Anxiety Disorder (GAD-7) scale by Spitzer et al [34] for participants to self-rate their anxiety symptoms. The GAD-7 questionnaire measures anxiety symptoms occurring within the past 2 weeks on a 4-point scale, ranging from 0 (not at all) to 3 (nearly every day). The 7 items were added together to provide a total anxiety score. The scores for the questionnaire ranged from 0 to 21. A score between 5 and 9 indicated mild anxiety, a score between 10 and 14 indicated moderate anxiety, and a score above 15 indicated severe anxiety [34]. In this study, the Cronbach α coefficients of the GAD-7 scale at baseline test and after the test were .794 and .795, respectively, indicating acceptable internal consistency.

#### Depressive Symptoms Measures

This study used the 10-item Major Depression Inventory (MDI) developed and validated by Bech et al [35] for participants to self-rate their depression symptoms. The MDI questionnaire measures depression symptoms occurring within the past 2 weeks on a 6-point rating scale ranging from 0 (no time) to 5 (all the time). Items 8 and 10 consist of 2-part questions (a and b), where the highest score between the 2 subquestions is used as the item score. The scores for this questionnaire ranged from 0 to 50. A score <20 indicates no or doubtful depression, a score between 21 and 25 indicates mild depression, a score between 26 and 30 indicates moderate depression, and a score >30 indicates severe depression [35]. In this study, the baseline test of the MDI had a Cronbach α of .770, whereas the posttest evaluation of the MDI had a Cronbach α of .773, indicating both having an acceptable internal consistency level.

### Data Analysis

Of the 77 participants who completed the preintervention and postintervention surveys, 3 were excluded from the final data analysis because of poor participation. The final data set included 46 participants from the WeActive intervention and 28 participants from the WeMindful intervention, for a total of 74 participants.

The preintervention and postintervention data were analyzed using SPSS 26 software (IBM Corporation), with statistical significance set at *P*<.05 for the tests. Of the 77 students who completed both the preintervention and postintervention surveys, 74 were included in the final analysis (46 from WeActive and 28 from WeMindful). Overall, three participants (2 from WeActive and 1 from WeMindful) were excluded from the final analysis because of a lack of adequate participation. The mean scores of the GAD-7 and MDI tests were compiled and used for dependent variable analysis. Descriptive statistics of each intervention at baseline were calculated, and 2-tailed independent *t* tests were used to determine any significant differences in the dependent variables of each intervention group. A mixed-design analysis of variance repeated measure was used to examine the effect of either intervention group on depression and anxiety measures. Depression and anxiety scores were the main dependent variables in this study, with univariate tests separately for each variable. In this study, the between-factor was the analysis of the WeActive intervention group against the WeMindful intervention group. The within-subject factor was the comparison of the preintervention test against the postintervention test. In the analysis of covariance (ANCOVA) tests, if sphericity was violated, the Greenhouse-Geisser correction was applied. The results showed no significant differences in the preintervention mean scores for X3month_exercise, X3month_yoga, MDI score, GAD-7 score, education, race, and age, with the only significant difference between groups coming from the X3month_therapist variable (*F*_1_=15.083; *P*=.01). Therefore, ANCOVA was used to determine the intervention effects on anxiety and depression while controlling for covariates. The between-subject factor for analysis was the intervention group, comparing the WeActive group with the WeMindful group, and the within-subject factor was time, comparing the baseline test scores with the posttest scores.

### Ethics Statement

This study was reviewed and approved by the University Institutional Review Board-Health Sciences and Behavioral Sciences (IRB-HSBS) (HUM00189120). We conducted this study in accordance with the principles of the Declaration of Helsinki. All participants in the study have provided written informed consent to participate in the study.

## Results

### Overview

The results showed significant decreases in depression symptoms in both intervention groups as a main effect of time. Anxiety symptoms decreased marginally in both groups. There were no significant effects of group or of time and group in affecting anxiety or depression symptoms.

### Baseline Characteristics

The final WeActive group consisted of 48 participants, with a mean age of 23.02 (SD 4.83) years. The final WeMindful group consisted of 29 participants, with a mean age of 24.31 (SD 7.48) years. Both groups had similar educational and racial distributions. A summary of the descriptive statistics is presented in Table 1**.**

Table 2 displays the baseline score difference between the 2 intervention groups.

**Table 1:** Descriptive statistics of baseline characteristics of all participants.

Characteristics			Values
**Sex, n (%)**			
	Female	63 (85.1)	
	Male	7 (9.5)	
	Nonbinary	4 (5.4)	
**Education, n (%)**			
	Freshman	8 (10.7)	
	Sophomore	9 (12)	
	Junior	15 (20)	
	Senior	14 (18.7)	
	Masters	13 (17.3)	
	Doctoral	14 (18.7)	
	Professional	2 (2.7)	
**Race, n (%)**			
	African American	4 (5.4)	
	Asian	15 (20.3)	
	White	48 (64.9)	
	White, Asian	3 (4.1)	
	White, native	1 (1.4)	
	White, other	1 (1.4)	
	Other	2 (2.7)	
**Age (years), mean (SD; mean of SD)**			
	WeActive (n=46)	23.02 (4.833; 0.713)	
	WeMindful (n=29)	24.31 (7.479; 1.389)	
**X3month_exercise^a^, mean (SD; mean of SD)**			
	WeActive (n=46)	0.47 (0.504; 0.072)	
	WeMindful (n=29)	0.38 (0.494; 0.092)	
**X3month_yoga^b^, mean (SD; mean of SD)**			
	WeActive (n=46)	0.16 (0.373; 0.053)	
	WeMindful (n=29)	0.24 (0.435; 0.081)	
**X3month_therapist^c^, mean (SD; mean of SD)**			
	WeActive (n=46)	0.20 (0.407; 0.058)	
	WeMindful (n=29)	0.48 (0.509; 0.094)	

^a^X3month_exercise: number of exercise sessions in the previous 3 months.^b^X3month_yoga: number of yoga sessions in the previous 3 months.^c^X3month_therapist: number of therapist visits in the previous 3 months.

**Table 2:** Independent t tests of baseline difference between the WeActive and the WeMindful groups.

Equal variances assumed	*t* test (*df*)	*P* value (2-tailed)
Age (years)	−0.908 (73)	.37
Race	−1.077 (72)	.29
Education	0.299 (73)	.77
X3month_exercise^a^	0.768 (76)	.45
X3month_yoga^b^	−0.839 (76)	.40
X3month_therapist^c^	−2.660 (76)	.01^d^
Health_rating	−0.610 (76)	.54
PreGADtotal^e^	−0.941 (76)	.35
PreMDItotal^f^	−0.509 (76)	.61

^a^X3month_exercise=number of exercise sessions in the previous 3 months.

^b^X3month_yoga=number of yoga sessions in the previous 3 months.

^c^X3month_therapist=number of therapist visits in the previous 3 months.

^d^*P*<.01.

^e^PreGADtotal=baseline total Generalized Anxiety Disorder.

^f^PreMDItotal=baseline total Major Depression Inventory.

As seen in Table 2, the mean scores of depression in both groups were similar, with the WeActive mean scores of 16.89 falling slightly lower than that of WeMindful at 17.75. Both scores are in the nondepressed classification range through the MDI, which is classified as a score under 20. According to the MDI criteria, both groups’ scores indicated *no or doubtful* levels of depression at baseline. Both groups presented similar scores in the anxiety measure, with WeActive at 7.15 and WeMindful at 7.79. These scores placed both groups within the *mild* anxiety range (score between 5 and 9) through the GAD-7. The mean scores of an overall health rating, 3-month history of exercise, and 3-month history of yoga were similar across both groups. Only the measure of the past 3-month therapist visits differed to a notable extent, with WeMindful averaging a higher score at 0.48 compared with the 0.2 therapist visit average for WeActive.

The independent sample *t* tests revealed no significant baseline differences in the outcome variables and demographic variables between the 2 groups (age: *P*=.41; race: *P*=.29; education: *P*=.77; physical activity: *P*=.36; anxiety: *P*=.32; depression: *P*=.60). With regard to the past 3-month therapist visit measure, the WeMindful group had a significantly higher mean average visit number than the WeActive group (*t*_1_=−2.660; *P*=.01). This baseline difference was controlled for when conducting repeated measures ANCOVA.

### Intervention Effects on Anxiety and Depression

Table 3 presents the baseline and posttest scores of anxiety and depression by group, whereas Table 4 displays the ANCOVA repeated measure results for anxiety and depression.

**Table 3:** Baseline and posttest scores of anxiety and depression.

			Values, mean (SD)
**Baseline anxiety**			
	WeActive	7.152 (5.517)	
	WeMindful	7.786 (4.475)	
**Posttest anxiety**			
	WeActive	6.652 (5.225)	
	WeMindful	6.429 (4.710)	
**Baseline depression**			
	WeActive	16.89 (12.97)	
	WeMindful	17.75 (10.77)	
**Posttest depression**			
	WeActive	13.57 (10.98)	
	WeMindful	16.89 (12.61)	

**Table 4:** Results of analyses of variance with repeated measures for anxiety and depression.

Effects	Anxiety				Depression		
	*F* test (*df*)	*P* value	*η* ^2^	*F* test (*df*)		*P* value	*η* ^2^
Time	0.485(1)	.07	0.047	0.892 (1)		.05^a^	0.052
Time×group	0.989 (1)	.32	0.014	0.914 (1)		.34	0.013
Group	0.423 (1)	.52	0.006	0.001 (1)		.98	0.000
X3_month_therapist	8.63 (1)	.004^b^	0.108	7.007 (1)		.01^b^	0.090

^a^*P*<.05.

^b^*P*<.01.

For the anxiety measure, while controlling for the past 3-month therapist visit, the results of ANCOVA with repeated measures indicated no significant main effect of time and group and no significant interaction between time and group. However, the main effect of time was close to a significant level (*F*=3.485; *P*=.07; *η*^2^=0.047), indicating that the 2 groups had a marginally significant decrease in anxiety over time. The resulting GAD-7 classification for both groups after the intervention was still considered within the *mild* range for anxiety severity. The results also showed that the past 3-month therapist visit was a significant covariate of anxiety (*F*=8.629; *P*=.004; *η*^2^=0.108).

With regard to the depression measure, the results of ANCOVA with repeated measures yielded a significant main effect of time (*F*=3.892; *P*=.05; *η*^2^=0.052), while controlling for the past 3-month therapist visit. The results indicated that the 2 groups showed a significant reduction in depressive symptoms from baseline to after the intervention. In contrast, the ANCOVA with repeated measures revealed no significant main effect of group and no significant interaction effect of time with group. The resulting MDI classification for both groups after the intervention was still considered within the *no or doubtful* range for depression severity. Similarly, the 3-month therapist visit was a significant covariate for depression (*F*=7.007; *P=*.01; *η*^2^=0.090).

## Discussion

### Principal Findings

This study aims to examine the effects of a web-based aerobic resistance exercise (WeActive) intervention and web-based yoga mindfulness (WeMindful) intervention on college students’ anxiety and depression levels. Both the 8-week interventions were hypothesized to have positive effects on anxiety and depression, with greater positive effects predicted in the WeActive group. As expected, the participants in the 2 groups experienced a significant decrease in depression scores over the 8-week period. Although not statistically significant, the participants experienced a decrease in anxiety scores as well. Contrary to our second hypothesis, there were no significant interactions between group and time, indicating that the WeActive group did not experience greater improvements in mental health scores than the WeMindful group over time.

The decrease in depression scores across both intervention groups in our study aligns with results from previous mindfulness interventions [12,20-23] and physical activity interventions [5,11-18]. The connection behind depression reduction and mindfulness exercises is thought to be caused by a variety of factors. With regard to emotional regulation, mindfulness activities interact directly and indirectly with mechanisms such as rumination and suppression, which act as mediators in the development of both depression and anxiety [36]. At the biological level, cortisol levels have been found to be higher in depressed individuals than in nondepressed individuals, with mindfulness activities such as yoga showing positive effects in reducing cortisol and improving depression measures [37]. Similar to the cortisol-related depression reduction mechanism seen with the use of mindfulness interventions, a reduction in depression due to increased physical activity has been linked to biological factors, such as neurogenesis in the hippocampus, acting similarly to antidepressant drugs [38], and through the regulation of stress hormone release through the hypothalamic-pituitary-adrenal axis [39]. With the implementation of both aerobic and resistance training interventions, some psychosocial symptoms of depression, such as negative thinking and self-efficacy, have been found to directly decrease, which in turn reduces depression severity [39]. It is likely that our WeActive and WeMindful interventions acted across a multitude of depression-meditating mechanisms, although the intervention sessions or length of intervention did not measure for biological changes in participants in either intervention group.

It is important to note that the participants in both groups experienced a marginally significant decrease in anxiety, a result that mostly supported our original hypothesis. Previous research that has examined the relationship between anxiety and either mindfulness or aerobic resistance exercise has displayed mixed results. Although most studies reveal a significant positive relationship [12-14,21-23], some studies show no significant interaction between the intervention and anxiety measure [16,20,29]. In a study by Bosso [30], inadequate participant adherence rates likely caused this lack of significance, with the anxiety measure in this study displaying a dose-dependent response to mindfulness activities. In addition, both the Bosso [30] and Conder [29] studies discovered that shortened intervention duration or low frequency likely limited the intervention effectiveness in reducing anxiety symptoms. Johnston et al [16] suggested that the anxiety levels in their study were likely influenced by external variables, such as increasing college demand over the course of a semester [16]. It is possible that our study had an insufficient frequency to produce significant anxiety changes. It is also likely that the onset of the college final examination period toward the end of the study greatly affected the overall anxiety levels of the participants. Although the results of our study do not indicate statistical significance, the mean anxiety scores across both interventions still notably decreased over time, showing the potential benefit of both the WeActive and WeMindful interventions.

Contrary to our expectations, there was no statistical significance in the group and time interactions, meaning neither the WeActive nor WeMindful group showed significant differences in depression or anxiety changes over time. Of the few studies that have compared the mental health effects of mindfulness exercises with aerobic or resistance exercises, only van der Zwan et al [31] showed similar results across both intervention groups, as in our study [31]. In the study by van der Zwan et al [31], both mindfulness exercise and aerobic exercise interventions showed significant reductions in anxiety and depression scores. As no research on this topic has been conducted in a pandemic environment, there are no definitive reasons yet established on why both intervention types displayed similar results. It is possible that the recent and collective changes in lifestyle may have contributed to the statistically equal impact of both the mindfulness exercise and aerobic resistance exercise interventions, suggesting that both interventions are effective in combating pandemic lifestyle–related depression symptoms. It is necessary to note that despite the presence of academic stressors such as final examinations and learning-related issues on the web, the students in both intervention groups showed a significant reduction in depression scores. This finding suggests that both aerobic and resistance training exercises as well as mindful exercises may be beneficial in buffering academic stress–related depression.

### Limitations

This study has several limitations. First, although initial participant interest was high, most interested potential participants decided not to proceed with the study. This limitation was likely caused by the completely web-based nature of this study, which prevented opportunities to connect more directly with participants and by the increasingly busy period of the school semester during which this study was conducted. The stressful nature of college classes, where stress often builds as the semester goes on, may have greatly hindered some of the students’ participation, as higher levels of stress related to schoolwork could have reduced the free time these participants had to complete the study. Second, the demographics of participants may have interfered with obtaining widely applicable findings, as most participants identified as female. Previous research has shown that females tend to report higher rates and severity of anxiety and depression symptoms than males [1,2]. Third, there may have been a ceiling effect present in the improvements of each group’s anxiety and depression scores, as both groups scored in the lowest severity categories of each disorder at baseline evaluation (*no or doubtful* for depression and *mild* for anxiety). These low baseline scores, indicating a low prevalence of anxiety and depression, may have prevented both the WeActive and WeMindful groups from showing larger improvements. A future study that screens more selectively for individuals experiencing higher levels of symptoms may show a greater effect. Finally, as this study was conducted during the midpandemic in a completely web-based environment, it is possible that some participants may have experienced *Zoom fatigue*, a state of exhaustion from overuse of internet web-based conferencing [40]. As school, work, and social settings had shifted to a primarily web-based setting for many students, the use of web-based interventions may have had effects opposite to what had been intended; instead of providing an accessible mindfulness exercise or physical activity option, the intervention setting could have been perceived as a deterrent or a reason for participant dropout. Future research on the topic of web-based mental health interventions would benefit from more gender-equal participant recruitment and distribution, possibly by comparing an in-person intervention setting with a web-based setting. In addition, future studies of mental health interventions in students could use different strategies to motivate and communicate with participants to reduce the participant attrition rate.

### Conclusions

Our study showed that the participants in both the WeActive and WeMindful groups experienced significantly decreased depression symptoms over time. This decrease in depression occurred despite the stressors of upcoming final examinations for all participants. Anxiety symptoms decreased but did not reach a statistically significant level. Both web-based aerobic resistance training and web-based yoga mindfulness exercises seem to be effective in buffering mental health distress, especially with depression. Future research should focus on examining the effects of web-based environments against in-person environments for reducing mental health disorder severity or symptoms in college students. In addition, as this study used multiple modalities in both aerobic resistance training and yoga mindfulness exercise interventions, future research could benefit from exploring single modality interventions, such as aerobic exercise compared with resistance training or yoga compared with meditation. This study suggests that both the combination of aerobic resistance training and the combination of yoga mindfulness exercises are associated with a reduction in college student depression scores over an 8-week period. As the findings from our study indicate the potential efficacy of WeActive and WeMindful interventions in improving student mental health measures, this study design could be a useful resource for colleges and mental health treatment providers.

## Appendix

Multimedia Appendix 1 CONSORT eHealth Checklist (V 1.6.1).

## Abbreviations

ANCOVA: analysis of covariance
CONSORT: Consolidated Standards of Reporting Trials
MDI: Major Depression Inventory
MVC: mindfulness virtual community
