# Supplementary material for: The Impact of Web-Based Physical Activity Interventions on Depression and Anxiety Among College Students: Randomized Experimental Trial
Source: JMIR Form Res. 2022 Apr 1;6(4):e31839. doi: 10.2196/31839 (PMC9015744; doi:10.2196/31839)
Supplement: Multimedia Appendix 1 [file formative_v6i4e31839_app1.pdf]

CONSORT-EHEALTH (V 1.6.1) - Submission/Publication Form

The CONSORT-EHEALTH checklist is intended for authors of randomized trials evaluating web-based and Internet-based applications/interventions, including mobile interventions, electronic games (incl multiplayer games), social media, certain telehealth applications, and other interactive and/or networked electronic applications. Some of the items (e.g. all subitems under item 5 - description of the intervention) may also be applicable for other study designs.

The goal of the CONSORT EHEALTH checklist and guideline is to be
a) a guide for reporting for authors of RCTs,
b) to form a basis for appraisal of an ehealth trial (in terms of validity)

CONSORT-EHEALTH items/subitems are MANDATORY reporting items for studies published in the Journal of Medical Internet Research and other journals / scientific societies endorsing the checklist.

Items numbered 1., 2., 3., 4a., 4b etc are original CONSORT or CONSORT-NPT (non-pharmacologic treatment) items.
Items with Roman numerals (i., ii, iii, iv etc.) are CONSORT-EHEALTH extensions/clarifications.

As the CONSORT-EHEALTH checklist is still considered in a formative stage, we would ask that you also RATE ON A SCALE OF 1-5 how important/useful you feel each item is FOR THE PURPOSE OF THE CHECKLIST and reporting guideline (optional).

Mandatory reporting items are marked with a red *.
In the textboxes, either copy & paste the relevant sections from your manuscript into this form - please include any quotes from your manuscript in QUOTATION MARKS,
or answer directly by providing additional information not in the manuscript, or elaborating on why the item was not relevant for this study.

YOUR ANSWERS WILL BE PUBLISHED AS A SUPPLEMENTARY FILE TO YOUR PUBLICATION IN JMIR AND ARE CONSIDERED PART OF YOUR PUBLICATION (IF ACCEPTED).
Please fill in these questions diligently. Information will not be copyedited, so please use proper spelling and grammar, use correct capitalization, and avoid abbreviations.

DO NOT FORGET TO SAVE AS PDF _AND_ CLICK THE SUBMIT BUTTON SO YOUR ANSWERS ARE IN OUR DATABASE !!!

Citation Suggestion (if you append the pdf as Appendix we suggest to cite this paper in the caption):
Eysenbach G, CONSORT-EHEALTH Group
CONSORT-EHEALTH: Improving and Standardizing Evaluation Reports of Web-based and Mobile Health Interventions
J Med Internet Res 2011;13(4):e126

URL: http://www.jmir.org/2011/4/e126/
doi: 10.2196/jmir.1923
PMID: 22209829

aawilliamsmurray@gmail.com (not shared) Switch account

Draft not saved

* Required

Your name *

First Last

Andy Murray

Primary Affiliation (short), City, Country *

University of Toronto, Toronto, Canada

University of Michigan, Ann Arbor MI, USA

Your e-mail address *

abc@gmail.com

migwetch@umich.edu

Title of your manuscript *

Provide the (draft) title of your manuscript.

The Impact of Web-Based Physical Activity Interventions on Depression and Anxiety Among College Students: Randomized Experimental Trial

Name of your App/Software/Intervention *

If there is a short and a long/alternate name, write the short name first and add the long name in brackets.

WeActive and WeMindful

Evaluated Version (if any)

e.g. "V1", "Release 2017-03-01", "Version 2.0.27913"

Your answer

Language(s) *

What language is the intervention/app in? If multiple languages are available, separate by comma (e.g. "English, French")

English

URL of your Intervention Website or App

e.g. a direct link to the mobile app on app in appstore (itunes, Google Play), or URL of the website. If the intervention is a DVD or hardware, you can also link to an Amazon page.

Your answer

URL of an image/screenshot (optional)

Your answer

Accessibility *

Can an enduser access the intervention presently?

- (●) access is free and open
- ( ) access only for special usergroups, not open
- ( ) access is open to everyone, but requires payment/subscription/in-app purchases
- ( ) app/intervention no longer accessible
- ( ) Other:

Primary Medical Indication/Disease/Condition *

e.g. "Stress", "Diabetes", or define the target group in brackets after the condition, e.g. "Autism (Parents of children with)", "Alzheimers (Informal Caregivers of)"

Anxiety and Depression

Primary Outcomes measured in trial *

comma-separated list of primary outcomes reported in the trial

Anxiety and Depression symptoms and scale

Secondary/other outcomes

Are there any other outcomes the intervention is expected to affect?

Your answer

Recommended "Dose" *

What do the instructions for users say on how often the app should be used?

- ( ) Approximately Daily
- (x) Approximately Weekly
- ( ) Approximately Monthly
- ( ) Approximately Yearly
- ( ) "as needed"
- ( ) Other:

Approx. Percentage of Users (starters) still using the app as recommended after 3 months *

- (x) unknown / not evaluated
- ( ) 0-10%
- ( ) 11-20%
- ( ) 21-30%
- ( ) 31-40%
- ( ) 41-50%
- ( ) 51-60%
- ( ) 61-70%
- ( ) 71%-80%
- ( ) 81-90%
- ( ) 91-100%
- ( ) Other:

Overall, was the app/intervention effective? *

- ○ yes: all primary outcomes were significantly better in intervention group vs control
- ◉ partly: SOME primary outcomes were significantly better in intervention group vs control
- ○ no statistically significant difference between control and intervention
- ○ potentially harmful: control was significantly better than intervention in one or more outcomes
- ○ inconclusive: more research is needed
- ○ Other:

Article Preparation Status/Stage *

At which stage in your article preparation are you currently (at the time you fill in this form)

- ○ not submitted yet - in early draft status
- ○ not submitted yet - in late draft status, just before submission
- ○ submitted to a journal but not reviewed yet
- ○ submitted to a journal and after receiving initial reviewer comments
- ◉ submitted to a journal and accepted, but not published yet
- ○ published
- ○ Other:

Journal *

If you already know where you will submit this paper (or if it is already submitted), please provide the journal name (if it is not JMIR, provide the journal name under "other")

- ○ not submitted yet / unclear where I will submit this
- ○ Journal of Medical Internet Research (JMIR)
- ○ JMIR mHealth and UHealth
- ○ JMIR Serious Games
- ○ JMIR Mental Health
- ○ JMIR Public Health
- ◉ JMIR Formative Research
- ○ Other JMIR sister journal
- ○ Other:

Is this a full powered effectiveness trial or a pilot/feasibility trial? *

- ○ Pilot/feasibility
- ◉ Fully powered

Manuscript tracking number *

If this is a JMIR submission, please provide the manuscript tracking number under "other" (The ms tracking number can be found in the submission acknowledgement email, or when you login as author in JMIR. If the paper is already published in JMIR, then the ms tracking number is the four-digit number at the end of the DOI, to be found at the bottom of each published article in JMIR)

- ○ no ms number (yet) / not (yet) submitted to / published in JMIR
- ◉ Other: 31839

TITLE AND ABSTRACT

1a) TITLE: Identification as a randomized trial in the title

1a) Does your paper address CONSORT item 1a? *

I.e does the title contain the phrase "Randomized Controlled Trial"? (if not, explain the reason under "other")

- ○ yes
- ◉ Other: Randomized Experimental Trial

1a-i) Identify the mode of delivery in the title

Identify the mode of delivery. Preferably use “web-based” and/or “mobile” and/or “electronic game” in the title. Avoid ambiguous terms like “online”, “virtual”, “interactive”. Use “Internet-based” only if Intervention includes non-web-based Internet components (e.g. email), use “computer-based” or “electronic” only if offline products are used. Use “virtual” only in the context of “virtual reality” (3-D worlds). Use “online” only in the context of “online support groups”. Complement or substitute product names with broader terms for the class of products (such as “mobile” or “smart phone” instead of “iphone”), especially if the application runs on different platforms.

	1	2	3	4	5	
subitem not at all important	○	○	○	○	◉	essential

Clear selection

Does your paper address subitem 1a-i? *

Copy and paste relevant sections from manuscript title (include quotes in quotation marks "like this" to indicate direct quotes from your manuscript), or elaborate on this item by providing additional information not in the ms, or briefly explain why the item is not applicable/relevant for your study

The Impact of Web-Based Physical Activity Interventions on Depression and Anxiety Among College Students: Randomized Experimental Trial

1a-ii) Non-web-based components or important co-interventions in title

Mention non-web-based components or important co-interventions in title, if any (e.g., “with telephone support”).

	1	2	3	4	5	
subitem not at all important	◉	○	○	○	○	essential

Clear selection

Does your paper address subitem 1a-ii?

Copy and paste relevant sections from manuscript title (include quotes in quotation marks "like this" to indicate direct quotes from your manuscript), or elaborate on this item by providing additional information not in the ms, or briefly explain why the item is not applicable/relevant for your study

Study was exclusively web-based

1a-iii) Primary condition or target group in the title

Mention primary condition or target group in the title, if any (e.g., “for children with Type I Diabetes”)
Example: A Web-based and Mobile Intervention with Telephone Support for Children with Type I Diabetes: Randomized Controlled Trial

	1	2	3	4	5	
subitem not at all important	○	○	○	○	◉	essential

Clear selection

Does your paper address subitem 1a-iii? *

Copy and paste relevant sections from manuscript title (include quotes in quotation marks "like this" to indicate direct quotes from your manuscript), or elaborate on this item by providing additional information not in the ms, or briefly explain why the item is not applicable/relevant for your study

Depression and Anxiety are directly mentioned

1b) ABSTRACT: Structured summary of trial design, methods, results, and conclusions

NPT extension: Description of experimental treatment, comparator, care providers, centers, and blinding status.

1b-i) Key features/functionalities/components of the intervention and comparator in the METHODS section of the ABSTRACT

Mention key features/functionalities/components of the intervention and comparator in the abstract. If possible, also mention theories and principles used for designing the site. Keep in mind the needs of systematic reviewers and indexers by including important synonyms. (Note: Only report in the abstract what the main paper is reporting. If this information is missing from the main body of text, consider adding it)

	1	2	3	4	5	
subitem not at all important	○	○	○	○	●	essential

Clear selection

Does your paper address subitem 1b-i? *

Copy and paste relevant sections from the manuscript abstract (include quotes in quotation marks "like this" to indicate direct quotes from your manuscript), or elaborate on this item by providing additional information not in the ms, or briefly explain why the item is not applicable/relevant for your study

"The participants were 77 college students who anonymously completed a Qualtrics survey, including the Generalized Anxiety Disorder Scale and the Major Depression Inventory at baseline and after the intervention. Participants were randomly assigned to either the WeActive or WeMindful group and underwent two 30-minute web-based aerobic resistance exercise lessons or yoga mindfulness lessons per week for 8 weeks."

1b-ii) Level of human involvement in the METHODS section of the ABSTRACT

Clarify the level of human involvement in the abstract, e.g., use phrases like “fully automated” vs. “therapist/nurse/care provider/physician-assisted” (mention number and expertise of providers involved, if any). (Note: Only report in the abstract what the main paper is reporting. If this information is missing from the main body of text, consider adding it)

	1	2	3	4	5	
subitem not at all important	○	◉	○	○	○	essential

Clear selection

Does your paper address subitem 1b-ii?

Copy and paste relevant sections from the manuscript abstract (include quotes in quotation marks "like this" to indicate direct quotes from your manuscript), or elaborate on this item by providing additional information not in the ms, or briefly explain why the item is not applicable/relevant for your study

Human involvement is implied ("Participants were randomly assigned to either the WeActive or WeMindful group and underwent two 30-minute web-based aerobic resistance exercise lessons or yoga mindfulness lessons per week for 8 weeks")

1b-iii) Open vs. closed, web-based (self-assessment) vs. face-to-face assessments in the METHODS section of the ABSTRACT

Mention how participants were recruited (online vs. offline), e.g., from an open access website or from a clinic or a closed online user group (closed usergroup trial), and clarify if this was a purely web-based trial, or there were face-to-face components (as part of the intervention or for assessment). Clearly say if outcomes were self-assessed through questionnaires (as common in web-based trials). Note: In traditional offline trials, an open trial (open-label trial) is a type of clinical trial in which both the researchers and participants know which treatment is being administered. To avoid confusion, use “blinded” or “unblinded” to indicated the level of blinding instead of “open”, as “open” in web-based trials usually refers to “open access” (i.e. participants can self-enrol). (Note: Only report in the abstract what the main paper is reporting. If this information is missing from the main body of text, consider adding it)

	1	2	3	4	5	
subitem not at all important	◉	○	○	○	○	essential

Clear selection

Does your paper address subitem 1b-iii?

Copy and paste relevant sections from the manuscript abstract (include quotes in quotation marks "like this" to indicate direct quotes from your manuscript), or elaborate on this item by providing additional information not in the ms, or briefly explain why the item is not applicable/relevant for your study

Recruitment and assessment not mentioned in abstract due to character limits

1b-iv) RESULTS section in abstract must contain use data

Report number of participants enrolled/assessed in each group, the use/uptake of the intervention (e.g., attrition/adherence metrics, use over time, number of logins etc.), in addition to primary/secondary outcomes. (Note: Only report in the abstract what the main paper is reporting. If this information is missing from the main body of text, consider adding it)

	1	2	3	4	5	
subitem not at all important	○	○	○	○	◉	essential

Clear selection

Does your paper address subitem 1b-iv?

Copy and paste relevant sections from the manuscript abstract (include quotes in quotation marks "like this" to indicate direct quotes from your manuscript), or elaborate on this item by providing additional information not in the ms, or briefly explain why the item is not applicable/relevant for your study

"The results of analysis of covariance with repeated measures indicated that although not statistically significant, both groups showed a notable decrease in anxiety with a marginally significant main effect of time ($F1=3.485$; $P=.07$; $\eta2=0.047$) but no significant main effect of group and no significant interaction effect of time with group. The 2 intervention groups experienced a significant decrease in depression with the main effect of time ($F=3.892$; $P=.05$; $\eta2=0.052$). There was no significant main effect of group or interaction effect of time with group for depression"

1b-v) CONCLUSIONS/DISCUSSION in abstract for negative trials

Conclusions/Discussions in abstract for negative trials: Discuss the primary outcome - if the trial is negative (primary outcome not changed), and the intervention was not used, discuss whether negative results are attributable to lack of uptake and discuss reasons. (Note: Only report in the abstract what the main paper is reporting. If this information is missing from the main body of text, consider adding it)

	1	2	3	4	5	
subitem not at all important	○	○	○	○	●	essential

Clear selection

Does your paper address subitem 1b-v?

Copy and paste relevant sections from the manuscript abstract (include quotes in quotation marks "like this" to indicate direct quotes from your manuscript), or elaborate on this item by providing additional information not in the ms, or briefly explain why the item is not applicable/relevant for your study

"College students in both WeActive and WeMindful groups experienced a significant decrease in depression symptoms and a decrease, although not significant, in anxiety as well."

INTRODUCTION

2a) In INTRODUCTION: Scientific background and explanation of rationale

2a-i) Problem and the type of system/solution

Describe the problem and the type of system/solution that is object of the study: intended as stand-alone intervention vs. incorporated in broader health care program? Intended for a particular patient population? Goals of the intervention, e.g., being more cost-effective to other interventions, replace or complement other solutions? (Note: Details about the intervention are provided in “Methods” under 5)

	1	2	3	4	5	
subitem not at all important	○	○	○	○	●	essential

Clear selection

Does your paper address subitem 2a-i? *

Copy and paste relevant sections from the manuscript (include quotes in quotation marks "like this" to indicate direct quotes from your manuscript), or elaborate on this item by providing additional information not in the ms, or briefly explain why the item is not applicable/relevant for your study

"It has been well-documented that mental health issues are increasing within a college student population. This increase in mental health problems, such as anxiety and depression, seems to have been exacerbated by the onset of the COVID-19 pandemic"

2a-ii) Scientific background, rationale: What is known about the (type of) system

Scientific background, rationale: What is known about the (type of) system that is the object of the study (be sure to discuss the use of similar systems for other conditions/diagnoses, if appropiate), motivation for the study, i.e. what are the reasons for and what is the context for this specific study, from which stakeholder viewpoint is the study performed, potential impact of findings [2]. Briefly justify the choice of the comparator.

	1	2	3	4	5	
subitem not at all important	○	○	○	○	◉	essential

Clear selection

Does your paper address subitem 2a-ii? *

Copy and paste relevant sections from the manuscript (include quotes in quotation marks "like this" to indicate direct quotes from your manuscript), or elaborate on this item by providing additional information not in the ms, or briefly explain why the item is not applicable/relevant for your study

"In Italy, research has found that 27.8% of 400 analyzed college students and employees displayed notable depressive symptoms and 34.3% displayed notable anxiety symptoms [3]. In the United States, 44% of 195 interviewed college students reported an increase in depressive symptoms, whereas 71% of the same sample of students reported an increase in stress and anxiety symptoms [4]. In a meta-analysis examining the percentage increases in self-reported anxiety and depression worldwide, researchers found an increase in anxiety from 19% to 37% and an increase in depression from 21% to 54% after March 1, 2020"

2b) In INTRODUCTION: Specific objectives or hypotheses

Does your paper address CONSORT subitem 2b? *

Copy and paste relevant sections from the manuscript (include quotes in quotation marks "like this" to indicate direct quotes from your manuscript), or elaborate on this item by providing additional information not in the ms, or briefly explain why the item is not applicable/relevant for your study

"In addition, the impact of the COVID-19 pandemic on the average mental health status of college students may have complicated the ways in which mindfulness and physical activity interventions interact with anxiety and depression symptoms, furthering the need to understand the utility of these interventions. To the best of our knowledge, there is a lack of research on the effects of web-based aerobic and resistance exercises or yoga with mindfulness on college student depression and anxiety in a midpandemic environment. Therefore, this study aims to compare the effects of a web-based aerobic resistance exercise intervention (WeActive) and a web-based yoga mindfulness intervention (WeMindful) on depression and anxiety in college students during the winter semester of 2021"

METHODS

3a) Description of trial design (such as parallel, factorial) including allocation ratio

Does your paper address CONSORT subitem 3a? *

Copy and paste relevant sections from the manuscript (include quotes in quotation marks "like this" to indicate direct quotes from your manuscript), or elaborate on this item by providing additional information not in the ms, or briefly explain why the item is not applicable/relevant for your study

"As seen in Figure 1, after the pre-evaluation, 77 participants attended the 8-week intervention components and completed the postintervention test.... On the basis of our previous study’s adherence rate of >90% and an estimated program dropout rate of 10%, we needed to recruit a targeted sample size of 57 college students. Our sample size of 77 participants exceeded the required sample size of 57."

3b) Important changes to methods after trial commencement (such as eligibility criteria), with reasons

Does your paper address CONSORT subitem 3b? *

Copy and paste relevant sections from the manuscript (include quotes in quotation marks "like this" to indicate direct quotes from your manuscript), or elaborate on this item by providing additional information not in the ms, or briefly explain why the item is not applicable/relevant for your study

No important changes were made nor mentioned

3b-i) Bug fixes, Downtimes, Content Changes

Bug fixes, Downtimes, Content Changes: ehealth systems are often dynamic systems. A description of changes to methods therefore also includes important changes made on the intervention or comparator during the trial (e.g., major bug fixes or changes in the functionality or content) (5-iii) and other “unexpected events” that may have influenced study design such as staff changes, system failures/downtimes, etc. [2].

	1	2	3	4	5	
subitem not at all important	◉	○	○	○	○	essential

Clear selection

Does your paper address subitem 3b-i?

Copy and paste relevant sections from the manuscript (include quotes in quotation marks "like this" to indicate direct quotes from your manuscript), or elaborate on this item by providing additional information not in the ms, or briefly explain why the item is not applicable/relevant for your study

No fixes or content changes were made

4a) Eligibility criteria for participants

Does your paper address CONSORT subitem 4a? *

Copy and paste relevant sections from the manuscript (include quotes in quotation marks "like this" to indicate direct quotes from your manuscript), or elaborate on this item by providing additional information not in the ms, or briefly explain why the item is not applicable/relevant for your study

"The eligibility criteria included being at least 18 years of age, having current status as a student at the university, and having consistent access to the internet and to the web-based conferencing app Zoom (Zoom Video Communications)."

4a-i) Computer / Internet literacy

Computer / Internet literacy is often an implicit “de facto” eligibility criterion - this should be explicitly clarified.

	1	2	3	4	5	
subitem not at all important	○	○	○	○	◉	essential

Clear selection

Does your paper address subitem 4a-i?

Copy and paste relevant sections from the manuscript (include quotes in quotation marks "like this" to indicate direct quotes from your manuscript), or elaborate on this item by providing additional information not in the ms, or briefly explain why the item is not applicable/relevant for your study

"The eligibility criteria included being at least 18 years of age, having current status as a student at the university, and having consistent access to the internet and to the web-based conferencing app Zoom (Zoom Video Communications)."

4a-ii) Open vs. closed, web-based vs. face-to-face assessments:

Open vs. closed, web-based vs. face-to-face assessments: Mention how participants were recruited (online vs. offline), e.g., from an open access website or from a clinic, and clarify if this was a purely web-based trial, or there were face-to-face components (as part of the intervention or for assessment), i.e., to what degree got the study team to know the participant. In online-only trials, clarify if participants were quasi-anonymous and whether having multiple identities was possible or whether technical or logistical measures (e.g., cookies, email confirmation, phone calls) were used to detect/prevent these.

	1	2	3	4	5	
subitem not at all important	○	○	○	○	◉	essential

Clear selection

Does your paper address subitem 4a-ii? *

Copy and paste relevant sections from the manuscript (include quotes in quotation marks "like this" to indicate direct quotes from your manuscript), or elaborate on this item by providing additional information not in the ms, or briefly explain why the item is not applicable/relevant for your study

"Both intervention groups were asked to attend two 30-minute group exercise classes via Zoom twice per week. Participants opted to attend 1 synchronized Zoom exercise lesson and 1 asynchronized lesson or 2 asynchronized lessons that were Zoom-recorded and uploaded to the University Canvas study-related website. The asynchronous recording contained the same lesson content from the synchronous live session, meaning content was repeated for the second session each week. In addition, all participants were asked to attend synchronous, researcher-led peer coaching sessions that took place once every 2 weeks for 30 minutes."

4a-iii) Information giving during recruitment

Information given during recruitment. Specify how participants were briefed for recruitment and in the informed consent procedures (e.g., publish the informed consent documentation as appendix, see also item X26), as this information may have an effect on user self-selection, user expectation and may also bias results.

	1	2	3	4	5	
subitem not at all important	○	○	○	○	◉	essential

Clear selection

Does your paper address subitem 4a-iii?

Copy and paste relevant sections from the manuscript (include quotes in quotation marks "like this" to indicate direct quotes from your manuscript), or elaborate on this item by providing additional information not in the ms, or briefly explain why the item is not applicable/relevant for your study

"The intervention lasted for 8 weeks over the course of the winter 2021 semester. Before the intervention started, participants were given 1 week to complete a preintervention Qualtrics survey that included anxiety and depression measures. Immediately after the intervention period, the participants were again given 1 week to complete a postintervention Qualtrics survey that included the same anxiety and depression measures used in the preintervention survey.... All participants in the study have provided written informed consent to participate in the study."

4b) Settings and locations where the data were collected

Does your paper address CONSORT subitem 4b? *

Copy and paste relevant sections from the manuscript (include quotes in quotation marks "like this" to indicate direct quotes from your manuscript), or elaborate on this item by providing additional information not in the ms, or briefly explain why the item is not applicable/relevant for your study

"The participants were instructed to attend two 30-minute exercise classes via Zoom per week for 8 weeks. The class duration was set to 30 minutes to maximize participant retention while still allowing participants to achieve an amount of exercise that has been shown to be beneficial [17]. The lessons were taught by a student instructor who had previously taken the University of Michigan classes Methods of Instruction for Exercises and Fundamentals of Strength and Conditioning." "This study used the 7-item Generalized Anxiety Disorder (GAD-7) scale by Spitzer et al [34] for participants to self-rate their anxiety symptoms.... This study used the 10-item Major Depression Inventory (MDI) developed and validated by Bech et al [35] for participants to self-rate their depression symptoms."

4b-i) Report if outcomes were (self-)assessed through online questionnaires

Clearly report if outcomes were (self-)assessed through online questionnaires (as common in web-based trials) or otherwise.

	1	2	3	4	5	
subitem not at all important	○	○	○	○	●	essential

Clear selection

Does your paper address subitem 4b-i? *

Copy and paste relevant sections from the manuscript (include quotes in quotation marks "like this" to indicate direct quotes from your manuscript), or elaborate on this item by providing additional information not in the ms, or briefly explain why the item is not applicable/relevant for your study

"This study used the 7-item Generalized Anxiety Disorder (GAD-7) scale by Spitzer et al [34] for participants to self-rate their anxiety symptoms.... This study used the 10-item Major Depression Inventory (MDI) developed and validated by Bech et al [35] for participants to self-rate their depression symptoms."

4b-ii) Report how institutional affiliations are displayed

Report how institutional affiliations are displayed to potential participants [on ehealth media], as affiliations with prestigious hospitals or universities may affect volunteer rates, use, and reactions with regards to an intervention.(Not a required item – describe only if this may bias results)

	1	2	3	4	5	
subitem not at all important	◉	○	○	○	○	essential

Clear selection

Does your paper address subitem 4b-ii?

Copy and paste relevant sections from the manuscript (include quotes in quotation marks "like this" to indicate direct quotes from your manuscript), or elaborate on this item by providing additional information not in the ms, or briefly explain why the item is not applicable/relevant for your study

No bias from institutional affiliation of intervention, as all recruited participants attended the same university as the lab from which the intervention was developed

5) The interventions for each group with sufficient details to allow replication, including how and when they were actually administered

5-i) Mention names, credential, affiliations of the developers, sponsors, and owners

Mention names, credential, affiliations of the developers, sponsors, and owners [6] (if authors/evaluators are owners or developer of the software, this needs to be declared in a “Conflict of interest” section or mentioned elsewhere in the manuscript).

	1	2	3	4	5	
subitem not at all important	○	○	◉	○	○	essential

Clear selection

Does your paper address subitem 5-i?

Copy and paste relevant sections from the manuscript (include quotes in quotation marks "like this" to indicate direct quotes from your manuscript), or elaborate on this item by providing additional information not in the ms, or briefly explain why the item is not applicable/relevant for your study

"Both intervention groups were asked to attend two 30-minute group exercise classes via Zoom twice per week. Participants opted to attend 1 synchronized Zoom exercise lesson and 1 asynchronized lesson or 2 asynchronized lessons that were Zoom-recorded and uploaded to the University Canvas study-related website. The asynchronous recording contained the same lesson content from the synchronous live session, meaning content was repeated for the second session each week. In addition, all participants were asked to attend synchronous, researcher-led peer coaching sessions that took place once every 2 weeks for 30 minutes."

5-ii) Describe the history/development process

Describe the history/development process of the application and previous formative evaluations (e.g., focus groups, usability testing), as these will have an impact on adoption/use rates and help with interpreting results.

	1	2	3	4	5	
subitem not at all important	(●)	○	○	○	○	essential

Clear selection

Does your paper address subitem 5-ii?

Copy and paste relevant sections from the manuscript (include quotes in quotation marks "like this" to indicate direct quotes from your manuscript), or elaborate on this item by providing additional information not in the ms, or briefly explain why the item is not applicable/relevant for your study

Not applicable - interventions were developed by the researchers solely for use in this study

5-iii) Revisions and updating

Revisions and updating. Clearly mention the date and/or version number of the application/intervention (and comparator, if applicable) evaluated, or describe whether the intervention underwent major changes during the evaluation process, or whether the development and/or content was “frozen” during the trial. Describe dynamic components such as news feeds or changing content which may have an impact on the replicability of the intervention (for unexpected events see item 3b).

	1	2	3	4	5	
subitem not at all important	(●)	( )	( )	( )	( )	essential

Clear selection

Does your paper address subitem 5-iii?

Copy and paste relevant sections from the manuscript (include quotes in quotation marks "like this" to indicate direct quotes from your manuscript), or elaborate on this item by providing additional information not in the ms, or briefly explain why the item is not applicable/relevant for your study

Not applicable - no updates or revisions were made

5-iv) Quality assurance methods

Provide information on quality assurance methods to ensure accuracy and quality of information provided [1], if applicable.

	1	2	3	4	5	
subitem not at all important	( )	( )	( )	( )	(●)	essential

Clear selection

Does your paper address subitem 5-iv?

Copy and paste relevant sections from the manuscript (include quotes in quotation marks "like this" to indicate direct quotes from your manuscript), or elaborate on this item by providing additional information not in the ms, or briefly explain why the item is not applicable/relevant for your study

"The lessons were taught by a student instructor who had previously taken the University of Michigan classes Methods of Instruction for Exercises and Fundamentals of Strength and Conditioning. In addition, the WeActive student instructor was a certified strength and conditioning specialist and a graduate student with ≥5 years of experience in personal training.... The lessons were taught by a student instructor who had previously taken the University of Michigan classes Methods of Instruction for Exercises and Fundamentals of Strength and Conditioning. In addition, the WeMindful student instructor was a junior undergraduate student studying movement science with 3 years of experience teaching yoga and dance classes."

5-v) Ensure replicability by publishing the source code, and/or providing screenshots/screen-capture video, and/or providing flowcharts of the algorithms used

Ensure replicability by publishing the source code, and/or providing screenshots/screen-capture video, and/or providing flowcharts of the algorithms used. Replicability (i.e., other researchers should in principle be able to replicate the study) is a hallmark of scientific reporting.

	1	2	3	4	5	
subitem not at all important	(●)	( )	( )	( )	( )	essential

Clear selection

Does your paper address subitem 5-v?

Copy and paste relevant sections from the manuscript (include quotes in quotation marks "like this" to indicate direct quotes from your manuscript), or elaborate on this item by providing additional information not in the ms, or briefly explain why the item is not applicable/relevant for your study

Not applicable within the manuscript, however authors are available to provide exercise/mindfulness programming used during this intervention upon request from interested parties

5-vi) Digital preservation

Digital preservation: Provide the URL of the application, but as the intervention is likely to change or disappear over the course of the years; also make sure the intervention is archived (Internet Archive, webcitation.org, and/or publishing the source code or screenshots/videos alongside the article). As pages behind login screens cannot be archived, consider creating demo pages which are accessible without login.

	1	2	3	4	5	
subitem not at all important	◉	○	○	○	○	essential

Clear selection

Does your paper address subitem 5-vi?

Copy and paste relevant sections from the manuscript (include quotes in quotation marks "like this" to indicate direct quotes from your manuscript), or elaborate on this item by providing additional information not in the ms, or briefly explain why the item is not applicable/relevant for your study

Not applicable within the manuscript, however authors are available to provide exercise/mindfulness programming used during this intervention upon request from interested parties

5-vii) Access

Access: Describe how participants accessed the application, in what setting/context, if they had to pay (or were paid) or not, whether they had to be a member of specific group. If known, describe how participants obtained "access to the platform and Internet" [1]. To ensure access for editors/reviewers/readers, consider to provide a "backdoor" login account or demo mode for reviewers/readers to explore the application (also important for archiving purposes, see vi).

	1	2	3	4	5	
subitem not at all important	○	○	○	○	◉	essential

Clear selection

Does your paper address subitem 5-vii? *

Copy and paste relevant sections from the manuscript (include quotes in quotation marks "like this" to indicate direct quotes from your manuscript), or elaborate on this item by providing additional information not in the ms, or briefly explain why the item is not applicable/relevant for your study

"Both intervention groups were asked to attend two 30-minute group exercise classes via Zoom twice per week. Participants opted to attend 1 synchronized Zoom exercise lesson and 1 asynchronized lesson or 2 asynchronized lessons that were Zoom-recorded and uploaded to the University Canvas study-related website. The asynchronous recording contained the same lesson content from the synchronous live session, meaning content was repeated for the second session each week. In addition, all participants were asked to attend synchronous, researcher-led peer coaching sessions that took place once every 2 weeks for 30 minutes."

5-viii) Mode of delivery, features/functionalities/components of the intervention and comparator, and the theoretical framework

Describe mode of delivery, features/functionalities/components of the intervention and comparator, and the theoretical framework [6] used to design them (instructional strategy [1], behaviour change techniques, persuasive features, etc., see e.g., [7, 8] for terminology). This includes an in-depth description of the content (including where it is coming from and who developed it) [1],” whether [and how] it is tailored to individual circumstances and allows users to track their progress and receive feedback” [6]. This also includes a description of communication delivery channels and – if computer-mediated communication is a component – whether communication was synchronous or asynchronous [6]. It also includes information on presentation strategies [1], including page design principles, average amount of text on pages, presence of hyperlinks to other resources, etc. [1].

	1	2	3	4	5	
subitem not at all important	○	○	○	○	◉	essential

Clear selection

Does your paper address subitem 5-viii? *

Copy and paste relevant sections from the manuscript (include quotes in quotation marks "like this" to indicate direct quotes from your manuscript), or elaborate on this item by providing additional information not in the ms, or briefly explain why the item is not applicable/relevant for your study

"Both intervention groups were asked to attend two 30-minute group exercise classes via Zoom twice per week. Participants opted to attend 1 synchronized Zoom exercise lesson and 1 asynchronized lesson or 2 asynchronized lessons that were Zoom-recorded and uploaded to the University Canvas study-related website. The asynchronous recording contained the same lesson content from the synchronous live session, meaning content was repeated for the second session each week. In addition, all participants were asked to attend synchronous, researcher-led peer coaching sessions that took place once every 2 weeks for 30 minutes." "The objectives of the WeActive intervention were to help the participants engage in at least 150 minutes of moderate to vigorous physical activity per week and improve their abilities to achieve the recommended amount of weekly moderate to vigorous physical activity minutes throughout the intervention. The participants were instructed to attend two 30-minute exercise classes via Zoom per week for 8 weeks. The class duration was set to 30 minutes to maximize participant retention while still allowing participants to achieve an amount of exercise that has been shown to be beneficial " "The WeMindful group was instructed to participate in two 30-minute web-based yoga mindfulness classes per week for 8 weeks. The use of yoga, which incorporates movement with breathing and self-awareness, as a primary mindfulness exercise has been supported by previous research" "To facilitate the participants to attend the WeActive or WeMindful exercise lessons and to provide ongoing emotional, informational, and appraisal support for them, we offered all participants in both groups with four 30-minute peer coaching sessions conducted via Zoom, with 1 session every 2 weeks."

5-ix) Describe use parameters

Describe use parameters (e.g., intended “doses” and optimal timing for use). Clarify what instructions or recommendations were given to the user, e.g., regarding timing, frequency, heaviness of use, if any, or was the intervention used ad libitum.

	1	2	3	4	5	
subitem not at all important	○	○	○	○	◉	essential

Clear selection

Does your paper address subitem 5-ix?

Copy and paste relevant sections from the manuscript (include quotes in quotation marks "like this" to indicate direct quotes from your manuscript), or elaborate on this item by providing additional information not in the ms, or briefly explain why the item is not applicable/relevant for your study

"Both intervention groups were asked to attend two 30-minute group exercise classes via Zoom twice per week. Participants opted to attend 1 synchronized Zoom exercise lesson and 1 asynchronized lesson or 2 asynchronized lessons that were Zoom-recorded and uploaded to the University Canvas study-related website. The asynchronous recording contained the same lesson content from the synchronous live session, meaning content was repeated for the second session each week. In addition, all participants were asked to attend synchronous, researcher-led peer coaching sessions that took place once every 2 weeks for 30 minutes." "The objectives of the WeActive intervention were to help the participants engage in at least 150 minutes of moderate to vigorous physical activity per week and improve their abilities to achieve the recommended amount of weekly moderate to vigorous physical activity minutes throughout the intervention. The participants were instructed to attend two 30-minute exercise classes via Zoom per week for 8 weeks. The class duration was set to 30 minutes to maximize participant retention while still allowing participants to achieve an amount of exercise that has been shown to be beneficial " "The WeMindful group was instructed to participate in two 30-minute web-based yoga mindfulness classes per week for 8 weeks. The use of yoga, which incorporates movement with breathing and self-awareness, as a primary mindfulness exercise has been supported by previous research"

5-x) Clarify the level of human involvement

Clarify the level of human involvement (care providers or health professionals, also technical assistance) in the e-intervention or as co-intervention (detail number and expertise of professionals involved, if any, as well as “type of assistance offered, the timing and frequency of the support, how it is initiated, and the medium by which the assistance is delivered”. It may be necessary to distinguish between the level of human involvement required for the trial, and the level of human involvement required for a routine application outside of a RCT setting (discuss under item 21 – generalizability).

	1	2	3	4	5	
subitem not at all important	○	○	○	○	◉	essential

Clear selection

Does your paper address subitem 5-x?

Copy and paste relevant sections from the manuscript (include quotes in quotation marks "like this" to indicate direct quotes from your manuscript), or elaborate on this item by providing additional information not in the ms, or briefly explain why the item is not applicable/relevant for your study

"In addition, all participants were asked to attend synchronous, researcher-led peer coaching sessions that took place once every 2 weeks for 30 minutes.... the WeMindful student instructor was a junior undergraduate student studying movement science with 3 years of experience teaching yoga and dance classes.... lessons were taught by a student instructor who had previously taken the University of Michigan classes Methods of Instruction for Exercises and Fundamentals of Strength and Conditioning. In addition, the WeActive student instructor was a certified strength and conditioning specialist and a graduate student with ≥5 years of experience in personal training."

5-xi) Report any prompts/reminders used

Report any prompts/reminders used: Clarify if there were prompts (letters, emails, phone calls, SMS) to use the application, what triggered them, frequency etc. It may be necessary to distinguish between the level of prompts/reminders required for the trial, and the level of prompts/reminders for a routine application outside of a RCT setting (discuss under item 21 – generalizability).

	1	2	3	4	5	
subitem not at all important	○	○	○	○	○	essential

Does your paper address subitem 5-xi? *

Copy and paste relevant sections from the manuscript (include quotes in quotation marks "like this" to indicate direct quotes from your manuscript), or elaborate on this item by providing additional information not in the ms, or briefly explain why the item is not applicable/relevant for your study

"To facilitate the participants to attend the WeActive or WeMindful exercise lessons and to provide ongoing emotional, informational, and appraisal support for them, we offered all participants in both groups with four 30-minute peer coaching sessions conducted via Zoom, with 1 session every 2 weeks..... In addition, participants received encouraging messages on the web from the research team every Thursday, with content including praise and motivational phrasing."

5-xii) Describe any co-interventions (incl. training/support)

Describe any co-interventions (incl. training/support): Clearly state any interventions that are provided in addition to the targeted eHealth intervention, as ehealth intervention may not be designed as stand-alone intervention. This includes training sessions and support [1]. It may be necessary to distinguish between the level of training required for the trial, and the level of training for a routine application outside of a RCT setting (discuss under item 21 – generalizability.

	1	2	3	4	5	
subitem not at all important	○	○	○	○	◉	essential

Clear selection

Does your paper address subitem 5-xii? *

Copy and paste relevant sections from the manuscript (include quotes in quotation marks "like this" to indicate direct quotes from your manuscript), or elaborate on this item by providing additional information not in the ms, or briefly explain why the item is not applicable/relevant for your study

"To facilitate the participants to attend the WeActive or WeMindful exercise lessons and to provide ongoing emotional, informational, and appraisal support for them, we offered all participants in both groups with four 30-minute peer coaching sessions conducted via Zoom, with 1 session every 2 weeks. Each peer coaching session included experience and feedback sharing from participants, barrier or challenge reflections, and participant suggestions for class content. The focus of the first peer coaching session was on orienting and introducing participants to the session formatting, which included goal-setting prompts, self-regulation prompts, self-monitoring reflection, and time for social support. The second, third, and fourth peer coaching sessions delved deeper into obtaining participant feedback on any challenges or preferences they had during the main intervention group components, along with continued goal-setting, self-regulation, and self-monitoring exercises. The peer coaching sessions were led by 1 doctoral student and 1 other undergraduate student of the research team. In addition to peer coaching sessions, all participants were instructed to complete weekly journals that asked participants to provide their weekly attendance for both synchronous and asynchronous classes. In addition, participants received encouraging messages on the web from the research team every Thursday, with content including praise and motivational phrasing."

6a) Completely defined pre-specified primary and secondary outcome measures, including how and when they were assessed

Does your paper address CONSORT subitem 6a? *

Copy and paste relevant sections from the manuscript (include quotes in quotation marks "like this" to indicate direct quotes from your manuscript), or elaborate on this item by providing additional information not in the ms, or briefly explain why the item is not applicable/relevant for your study

"Anxiety Measure
This study used the 7-item Generalized Anxiety Disorder (GAD-7) scale by Spitzer et al [34] for participants to self-rate their anxiety symptoms. The GAD-7 questionnaire measures anxiety symptoms occurring within the past 2 weeks on a 4-point scale, ranging from 0 (not at all) to 3 (nearly every day). The 7 items were added together to provide a total anxiety score. The scores for the questionnaire ranged from 0 to 21. A score between 5 and 9 indicated mild anxiety, a score between 10 and 14 indicated moderate anxiety, and a score above 15 indicated severe anxiety [34]. In this study, the Cronbach α coefficients of the GAD-7 scale at baseline test and after the test were .794 and .795, respectively, indicating acceptable internal consistency.

Depressive Symptoms Measures
This study used the 10-item Major Depression Inventory (MDI) developed and validated by Bech et al [35] for participants to self-rate their depression symptoms. The MDI questionnaire measures depression symptoms occurring within the past 2 weeks on a 6-point rating scale ranging from 0 (no time) to 5 (all the time). Items 8 and 10 consist of 2-part questions (a and b), where the highest score between the 2 subquestions is used as the item score. The scores for this questionnaire ranged from 0 to 50. A score <20 indicates no or doubtful depression, a score between 21 and 25 indicates mild depression, a score between 26 and 30 indicates moderate depression, and a score >30 indicates severe depression [35]. In this study, the baseline test of the MDI had a Cronbach α of .770, whereas the posttest evaluation of the MDI had a Cronbach α of .773, indicating both having an acceptable internal consistency level."

6a-i) Online questionnaires: describe if they were validated for online use and apply CHERRIES items to describe how the questionnaires were designed/deployed

If outcomes were obtained through online questionnaires, describe if they were validated for online use and apply CHERRIES items to describe how the questionnaires were designed/deployed [9].

	1	2	3	4	5	
subitem not at all important	○	○	○	○	◉	essential

Clear selection

Does your paper address subitem 6a-i?

Copy and paste relevant sections from manuscript text

"Anxiety Measure
This study used the 7-item Generalized Anxiety Disorder (GAD-7) scale by Spitzer et al [34] for participants to self-rate their anxiety symptoms. The GAD-7 questionnaire measures anxiety symptoms occurring within the past 2 weeks on a 4-point scale, ranging from 0 (not at all) to 3 (nearly every day). The 7 items were added together to provide a total anxiety score. The scores for the questionnaire ranged from 0 to 21. A score between 5 and 9 indicated mild anxiety, a score between 10 and 14 indicated moderate anxiety, and a score above 15 indicated severe anxiety [34]. In this study, the Cronbach α coefficients of the GAD-7 scale at baseline test and after the test were .794 and .795, respectively, indicating acceptable internal consistency.

Depressive Symptoms Measures
This study used the 10-item Major Depression Inventory (MDI) developed and validated by Bech et al [35] for participants to self-rate their depression symptoms. The MDI questionnaire measures depression symptoms occurring within the past 2 weeks on a 6-point rating scale ranging from 0 (no time) to 5 (all the time). Items 8 and 10 consist of 2-part questions (a and b), where the highest score between the 2 subquestions is used as the item score. The scores for this questionnaire ranged from 0 to 50. A score <20 indicates no or doubtful depression, a score between 21 and 25 indicates mild depression, a score between 26 and 30 indicates moderate depression, and a score >30 indicates severe depression [35]. In this study, the baseline test of the MDI had a Cronbach α of .770, whereas the posttest evaluation of the MDI had a Cronbach α of .773, indicating both having an acceptable internal consistency level."

6a-ii) Describe whether and how "use" (including intensity of use/dosage) was defined/measured/monitored

Describe whether and how "use" (including intensity of use/dosage) was defined/measured/monitored (logins, logfile analysis, etc.). Use/adoption metrics are important process outcomes that should be reported in any ehealth trial.

	1	2	3	4	5	
subitem not at all important	○	○	○	○	○	essential

Does your paper address subitem 6a-ii?

Copy and paste relevant sections from manuscript text

"Of the 77 participants who completed the preintervention and postintervention surveys, 3 were excluded from the final data analysis because of poor participation. The final data set included 46 participants from the WeActive intervention and 28 participants from the WeMindful intervention, for a total of 74 participants."

6a-iii) Describe whether, how, and when qualitative feedback from participants was obtained

Describe whether, how, and when qualitative feedback from participants was obtained (e.g., through emails, feedback forms, interviews, focus groups).

	1	2	3	4	5	
subitem not at all important	(selected)	○	○	○	○	essential

Clear selection

Does your paper address subitem 6a-iii?

Copy and paste relevant sections from manuscript text

Not applicable - no standardized feedback obtained

6b) Any changes to trial outcomes after the trial commenced, with reasons

Does your paper address CONSORT subitem 6b? *

Copy and paste relevant sections from the manuscript (include quotes in quotation marks "like this" to indicate direct quotes from your manuscript), or elaborate on this item by providing additional information not in the ms, or briefly explain why the item is not applicable/relevant for your study

Yes - no changes to trial outcomes were made

7a) How sample size was determined

NPT: When applicable, details of whether and how the clustering by care provides or centers was addressed

7a-i) Describe whether and how expected attrition was taken into account when calculating the sample size

Describe whether and how expected attrition was taken into account when calculating the sample size.

	1	2	3	4	5	
subitem not at all important	○	○	○	○	◉	essential

Clear selection

Does your paper address subitem 7a-i?

Copy and paste relevant sections from manuscript title (include quotes in quotation marks "like this" to indicate direct quotes from your manuscript), or elaborate on this item by providing additional information not in the ms, or briefly explain why the item is not applicable/relevant for your study

Your answer

7b) When applicable, explanation of any interim analyses and stopping guidelines

Does your paper address CONSORT subitem 7b? *

Copy and paste relevant sections from the manuscript (include quotes in quotation marks "like this" to indicate direct quotes from your manuscript), or elaborate on this item by providing additional information not in the ms, or briefly explain why the item is not applicable/relevant for your study

"We calculated the study sample size with an effect size, Cohen d=0.80, on college students' anxiety [33], 2-tailed with an α level of .05 and a power of 0.80 using G*Power 3.1.9.7 software. The results showed that the total required sample size of the study was 52, with 26 students in each intervention group. On the basis of our previous study's adherence rate of >90% and an estimated program dropout rate of 10%, we needed to recruit a targeted sample size of 57 college students. Our sample size of 77 participants exceeded the required sample size of 57."

8a) Method used to generate the random allocation sequence

NPT: When applicable, how care providers were allocated to each trial group

Does your paper address CONSORT subitem 8a? *

Copy and paste relevant sections from the manuscript (include quotes in quotation marks "like this" to indicate direct quotes from your manuscript), or elaborate on this item by providing additional information not in the ms, or briefly explain why the item is not applicable/relevant for your study

"Participants were randomly assigned to either the WeActive or WeMindful group and underwent two 30-minute web-based aerobic resistance exercise lessons or yoga mindfulness lessons per week for 8 weeks"

8b) Type of randomisation; details of any restriction (such as blocking and block size)

Does your paper address CONSORT subitem 8b? *

Copy and paste relevant sections from the manuscript (include quotes in quotation marks "like this" to indicate direct quotes from your manuscript), or elaborate on this item by providing additional information not in the ms, or briefly explain why the item is not applicable/relevant for your study

Not mentioned in the manuscript

9) Mechanism used to implement the random allocation sequence (such as sequentially numbered containers), describing any steps taken to conceal the sequence until interventions were assigned

Does your paper address CONSORT subitem 9? *

Copy and paste relevant sections from the manuscript (include quotes in quotation marks "like this" to indicate direct quotes from your manuscript), or elaborate on this item by providing additional information not in the ms, or briefly explain why the item is not applicable/relevant for your study

Not mentioned in the manuscript

10) Who generated the random allocation sequence, who enrolled participants, and who assigned participants to interventions

Does your paper address CONSORT subitem 10? *

Copy and paste relevant sections from the manuscript (include quotes in quotation marks "like this" to indicate direct quotes from your manuscript), or elaborate on this item by providing additional information not in the ms, or briefly explain why the item is not applicable/relevant for your study

Not mentioned in the manuscript

11a) If done, who was blinded after assignment to interventions (for example, participants, care providers, those assessing outcomes) and how

NPT: Whether or not administering co-interventions were blinded to group assignment

11a-i) Specify who was blinded, and who wasn’t

Specify who was blinded, and who wasn’t. Usually, in web-based trials it is not possible to blind the participants [1, 3] (this should be clearly acknowledged), but it may be possible to blind outcome assessors, those doing data analysis or those administering co-interventions (if any).

	1	2	3	4	5	
subitem not at all important	◉	○	○	○	○	essential

Clear selection

Does your paper address subitem 11a-i? *

Copy and paste relevant sections from the manuscript (include quotes in quotation marks "like this" to indicate direct quotes from your manuscript), or elaborate on this item by providing additional information not in the ms, or briefly explain why the item is not applicable/relevant for your study

Not applicable - no blinding was needed for this study design

11a-ii) Discuss e.g., whether participants knew which intervention was the “intervention of interest” and which one was the “comparator”

Informed consent procedures (4a-ii) can create biases and certain expectations - discuss e.g., whether participants knew which intervention was the “intervention of interest” and which one was the “comparator”.

	1	2	3	4	5	
subitem not at all important	◉	○	○	○	○	essential

Clear selection

Does your paper address subitem 11a-ii?

Copy and paste relevant sections from the manuscript (include quotes in quotation marks "like this" to indicate direct quotes from your manuscript), or elaborate on this item by providing additional information not in the ms, or briefly explain why the item is not applicable/relevant for your study

Both interventions were considered primary interventions

11b) If relevant, description of the similarity of interventions

(this item is usually not relevant for ehealth trials as it refers to similarity of a placebo or sham intervention to a active medication/intervention)

Does your paper address CONSORT subitem 11b? *

Copy and paste relevant sections from the manuscript (include quotes in quotation marks "like this" to indicate direct quotes from your manuscript), or elaborate on this item by providing additional information not in the ms, or briefly explain why the item is not applicable/relevant for your study

"Both intervention groups were asked to attend two 30-minute group exercise classes via Zoom twice per week. Participants opted to attend 1 synchronized Zoom exercise lesson and 1 asynchronized lesson or 2 asynchronized lessons that were Zoom-recorded and uploaded to the University Canvas study-related website. The asynchronous recording contained the same lesson content from the synchronous live session, meaning content was repeated for the second session each week. In addition, all participants were asked to attend synchronous, researcher-led peer coaching sessions that took place once every 2 weeks for 30 minutes.

The intervention lasted for 8 weeks over the course of the winter 2021 semester. Before the intervention started, participants were given 1 week to complete a preintervention Qualtrics survey that included anxiety and depression measures. Immediately after the intervention period, the participants were again given 1 week to complete a postintervention Qualtrics survey that included the same anxiety and depression measures used in the preintervention survey."

12a) Statistical methods used to compare groups for primary and secondary outcomes

NPT: When applicable, details of whether and how the clustering by care providers or centers was addressed

Does your paper address CONSORT subitem 12a? *

Copy and paste relevant sections from the manuscript (include quotes in quotation marks "like this" to indicate direct quotes from your manuscript), or elaborate on this item by providing additional information not in the ms, or briefly explain why the item is not applicable/relevant for your study

"The preintervention and postintervention data were analyzed using SPSS 26 software (IBM Corporation), with statistical significance set at P<.05 for the tests. Of the 77 students who completed both the preintervention and postintervention surveys, 74 were included in the final analysis (46 from WeActive and 28 from WeMindful). Overall, three participants (2 from WeActive and 1 from WeMindful) were excluded from the final analysis because of a lack of adequate participation. The mean scores of the GAD-7 and MDI tests were compiled and used for dependent variable analysis. Descriptive statistics of each intervention at baseline were calculated, and 2-tailed independent t tests were used to determine any significant differences in the dependent variables of each intervention group. A mixed-design analysis of variance repeated measure was used to examine the effect of either intervention group on depression and anxiety measures. Depression and anxiety scores were the main dependent variables in this study, with univariate tests separately for each variable. In this study, the between-factor was the analysis of the WeActive intervention group against the WeMindful intervention group. The within-subject factor was the comparison of the preintervention test against the postintervention test. In the analysis of covariance (ANCOVA) tests, if sphericity was violated, the Greenhouse-Geisser correction was applied. The results showed no significant differences in the preintervention mean scores for X3month_exercise, X3month_yoga, MDI score, GAD-7 score, education, race, and age, with the only significant difference between groups coming from the X3month_therapist variable (F1=15.083; P=.01). Therefore, ANCOVA was used to determine the intervention effects on anxiety and depression while controlling for covariates. The between-subject factor for analysis was the intervention group, comparing the WeActive group with the WeMindful group, and the within-subject factor was time, comparing the baseline test scores with the posttest scores."

12a-i) Imputation techniques to deal with attrition / missing values

Imputation techniques to deal with attrition / missing values: Not all participants will use the intervention/comparator as intended and attrition is typically high in ehealth trials. Specify how participants who did not use the application or dropped out from the trial were treated in the statistical analysis (a complete case analysis is strongly discouraged, and simple imputation techniques such as LOCF may also be problematic [4]).

	1	2	3	4	5	
subitem not at all important	○	○	○	○	◉	essential

Clear selection

Does your paper address subitem 12a-i? *

Copy and paste relevant sections from the manuscript (include quotes in quotation marks "like this" to indicate direct quotes from your manuscript), or elaborate on this item by providing additional information not in the ms, or briefly explain why the item is not applicable/relevant for your study

"Of the 77 participants who completed the preintervention and postintervention surveys, 3 were excluded from the final data analysis because of poor participation. The final data set included 46 participants from the WeActive intervention and 28 participants from the WeMindful intervention, for a total of 74 participants."

12b) Methods for additional analyses, such as subgroup analyses and adjusted analyses

Does your paper address CONSORT subitem 12b? *

Copy and paste relevant sections from the manuscript (include quotes in quotation marks "like this" to indicate direct quotes from your manuscript), or elaborate on this item by providing additional information not in the ms, or briefly explain why the item is not applicable/relevant for your study

""The preintervention and postintervention data were analyzed using SPSS 26 software (IBM Corporation), with statistical significance set at P<.05 for the tests. Of the 77 students who completed both the preintervention and postintervention surveys, 74 were included in the final analysis (46 from WeActive and 28 from WeMindful). Overall, three participants (2 from WeActive and 1 from WeMindful) were excluded from the final analysis because of a lack of adequate participation. The mean scores of the GAD-7 and MDI tests were compiled and used for dependent variable analysis. Descriptive statistics of each intervention at baseline were calculated, and 2-tailed independent t tests were used to determine any significant differences in the dependent variables of each intervention group. A mixed-design analysis of variance repeated measure was used to examine the effect of either intervention group on depression and anxiety measures. Depression and anxiety scores were the main dependent variables in this study, with univariate tests separately for each variable. In this study, the between-factor was the analysis of the WeActive intervention group against the WeMindful intervention group. The within-subject factor was the comparison of the preintervention test against the postintervention test. In the analysis of covariance (ANCOVA) tests, if sphericity was violated, the Greenhouse-Geisser correction was applied. The results showed no significant differences in the preintervention mean scores for X3month_exercise, X3month_yoga, MDI score, GAD-7 score, education, race, and age, with the only significant difference between groups coming from the X3month_therapist variable (F1=15.083; P=.01). Therefore, ANCOVA was used to determine the intervention effects on anxiety and depression while controlling for covariates. The between-subject factor for analysis was the intervention group, comparing the WeActive group with the WeMindful group, and the within-subject factor was time, comparing the baseline test scores with the posttest scores.""

X26) REB/IRB Approval and Ethical Considerations [recommended as subheading under "Methods"] (not a CONSORT item)

X26-i) Comment on ethics committee approval

	1	2	3	4	5	
subitem not at all important	○	○	○	○	●	essential

Clear selection

Does your paper address subitem X26-i?

Copy and paste relevant sections from the manuscript (include quotes in quotation marks "like this" to indicate direct quotes from your manuscript), or elaborate on this item by providing additional information not in the ms, or briefly explain why the item is not applicable/relevant for your study

"This study was reviewed and approved by the University Institutional Review Board-Health Sciences and Behavioral Sciences (IRB-HSBS) (HUM00189120). We conducted this study in accordance with the principles of the Declaration of Helsinki. All participants in the study have provided written informed consent to participate in the study."

x26-ii) Outline informed consent procedures

Outline informed consent procedures e.g., if consent was obtained offline or online (how? Checkbox, etc.?), and what information was provided (see 4a-ii). See [6] for some items to be included in informed consent documents.

	1	2	3	4	5	
subitem not at all important	(●)	( )	( )	( )	( )	essential

Clear selection

Does your paper address subitem X26-ii?

Copy and paste relevant sections from the manuscript (include quotes in quotation marks "like this" to indicate direct quotes from your manuscript), or elaborate on this item by providing additional information not in the ms, or briefly explain why the item is not applicable/relevant for your study

Consent procedures were not outlined in this manuscript, but were conducted electronically

X26-iii) Safety and security procedures

Safety and security procedures, incl. privacy considerations, and any steps taken to reduce the likelihood or detection of harm (e.g., education and training, availability of a hotline)

	1	2	3	4	5	
subitem not at all important	( )	( )	( )	( )	(●)	essential

Clear selection

Does your paper address subitem X26-iii?

Copy and paste relevant sections from the manuscript (include quotes in quotation marks "like this" to indicate direct quotes from your manuscript), or elaborate on this item by providing additional information not in the ms, or briefly explain why the item is not applicable/relevant for your study

" Each peer coaching session included experience and feedback sharing from participants, barrier or challenge reflections, and participant suggestions for class content. The focus of the first peer coaching session was on orienting and introducing participants to the session formatting, which included goal-setting prompts, self-regulation prompts, self-monitoring reflection, and time for social support. The second, third, and fourth peer coaching sessions delved deeper into obtaining participant feedback on any challenges or preferences they had during the main intervention group components, along with continued goal-setting, self-regulation, and self-monitoring exercises"

RESULTS

13a) For each group, the numbers of participants who were randomly assigned, received intended treatment, and were analysed for the primary outcome

NPT: The number of care providers or centers performing the intervention in each group and the number of patients treated by each care provider in each center

Does your paper address CONSORT subitem 13a? *

Copy and paste relevant sections from the manuscript (include quotes in quotation marks "like this" to indicate direct quotes from your manuscript), or elaborate on this item by providing additional information not in the ms, or briefly explain why the item is not applicable/relevant for your study

"The final WeActive group consisted of 48 participants, with a mean age of 23.02 (SD 4.83) years. The final WeMindful group consisted of 29 participants, with a mean age of 24.31 (SD 7.48) years. Both groups had similar educational and racial distributions... As seen in Table 2, the mean scores of depression in both groups were similar, with the WeActive mean scores of 16.89 falling slightly lower than that of WeMindful at 17.75. Both scores are in the nondepressed classification range through the MDI, which is classified as a score under 20. According to the MDI criteria, both groups' scores indicated no or doubtful levels of depression at baseline. Both groups presented similar scores in the anxiety measure, with WeActive at 7.15 and WeMindful at 7.79. These scores placed both groups within the mild anxiety range (score between 5 and 9) through the GAD-7. The mean scores of an overall health rating, 3-month history of exercise, and 3-month history of yoga were similar across both groups. Only the measure of the past 3-month therapist visits differed to a notable extent, with WeMindful averaging a higher score at 0.48 compared with the 0.2 therapist visit average for WeActive."

13b) For each group, losses and exclusions after randomisation, together with reasons

Does your paper address CONSORT subitem 13b? (NOTE: Preferably, this is shown in a CONSORT flow diagram) *

Copy and paste relevant sections from the manuscript (include quotes in quotation marks "like this" to indicate direct quotes from your manuscript), or elaborate on this item by providing additional information not in the ms, or briefly explain why the item is not applicable/relevant for your study

Yes - available in CONSORT diagram (Fig 1)

13b-i) Attrition diagram

Strongly recommended: An attrition diagram (e.g., proportion of participants still logging in or using the intervention/comparator in each group plotted over time, similar to a survival curve) or other figures or tables demonstrating usage/dose/engagement.

	1	2	3	4	5	
subitem not at all important	◉	○	○	○	○	essential

Clear selection

Does your paper address subitem 13b-i?

Copy and paste relevant sections from the manuscript or cite the figure number if applicable (include quotes in quotation marks "like this" to indicate direct quotes from your manuscript), or elaborate on this item by providing additional information not in the ms, or briefly explain why the item is not applicable/relevant for your study

Not provided for this manuscript, but attrition was described

14a) Dates defining the periods of recruitment and follow-up

Does your paper address CONSORT subitem 14a? *

Copy and paste relevant sections from the manuscript (include quotes in quotation marks "like this" to indicate direct quotes from your manuscript), or elaborate on this item by providing additional information not in the ms, or briefly explain why the item is not applicable/relevant for your study

Recruitment during "winter 2021" - no follow-up

14a-i) Indicate if critical “secular events” fell into the study period

Indicate if critical “secular events” fell into the study period, e.g., significant changes in Internet resources available or “changes in computer hardware or Internet delivery resources”

	1	2	3	4	5	
subitem not at all important	◉	○	○	○	○	essential

Clear selection

Does your paper address subitem 14a-i?

Copy and paste relevant sections from the manuscript (include quotes in quotation marks "like this" to indicate direct quotes from your manuscript), or elaborate on this item by providing additional information not in the ms, or briefly explain why the item is not applicable/relevant for your study

No secular events occurred

14b) Why the trial ended or was stopped (early)

Does your paper address CONSORT subitem 14b? *

Copy and paste relevant sections from the manuscript (include quotes in quotation marks "like this" to indicate direct quotes from your manuscript), or elaborate on this item by providing additional information not in the ms, or briefly explain why the item is not applicable/relevant for your study

The trial ended upon completion of the 8-week interventions

15) A table showing baseline demographic and clinical characteristics for each group

NPT: When applicable, a description of care providers (case volume, qualification, expertise, etc.) and centers (volume) in each group

Does your paper address CONSORT subitem 15? *

Copy and paste relevant sections from the manuscript (include quotes in quotation marks "like this" to indicate direct quotes from your manuscript), or elaborate on this item by providing additional information not in the ms, or briefly explain why the item is not applicable/relevant for your study

Included in the manuscript (Table 1, 2)

15-i) Report demographics associated with digital divide issues

In ehealth trials it is particularly important to report demographics associated with digital divide issues, such as age, education, gender, social-economic status, computer/Internet/ehealth literacy of the participants, if known.

	1	2	3	4	5	
subitem not at all important	◉	○	○	○	○	essential

Clear selection

Does your paper address subitem 15-i? *

Copy and paste relevant sections from the manuscript (include quotes in quotation marks "like this" to indicate direct quotes from your manuscript), or elaborate on this item by providing additional information not in the ms, or briefly explain why the item is not applicable/relevant for your study

Not applicable (no digital divide involved in this study), but demographics provided in Table 1

16) For each group, number of participants (denominator) included in each analysis and whether the analysis was by original assigned groups

16-i) Report multiple “denominators” and provide definitions

Report multiple “denominators” and provide definitions: Report N’s (and effect sizes) “across a range of study participation [and use] thresholds” [1], e.g., N exposed, N consented, N used more than x times, N used more than y weeks, N participants “used” the intervention/comparator at specific pre-defined time points of interest (in absolute and relative numbers per group). Always clearly define “use” of the intervention.

	1	2	3	4	5	
subitem not at all important	○	○	○	○	◉	essential

Clear selection

Does your paper address subitem 16-i? *

Copy and paste relevant sections from the manuscript (include quotes in quotation marks "like this" to indicate direct quotes from your manuscript), or elaborate on this item by providing additional information not in the ms, or briefly explain why the item is not applicable/relevant for your study

The final WeActive group consisted of 48 participants, with a mean age of 23.02 (SD 4.83) years. The final WeMindful group consisted of 29 participants, with a mean age of 24.31 (SD 7.48) years.

16-ii) Primary analysis should be intent-to-treat

Primary analysis should be intent-to-treat, secondary analyses could include comparing only “users”, with the appropriate caveats that this is no longer a randomized sample (see 18-i).

	1	2	3	4	5	
subitem not at all important	○	○	○	○	◉	essential

Clear selection

Does your paper address subitem 16-ii?

Copy and paste relevant sections from the manuscript (include quotes in quotation marks "like this" to indicate direct quotes from your manuscript), or elaborate on this item by providing additional information not in the ms, or briefly explain why the item is not applicable/relevant for your study

"For the anxiety measure, while controlling for the past 3-month therapist visit, the results of ANCOVA with repeated measures indicated no significant main effect of time and group and no significant interaction between time and group. However, the main effect of time was close to a significant level ($F=3.485$; $P=.07$; $\eta^2=0.047$), indicating that the 2 groups had a marginally significant decrease in anxiety over time. The resulting GAD-7 classification for both groups after the intervention was still considered within the mild range for anxiety severity. The results also showed that the past 3-month therapist visit was a significant covariate of anxiety ($F=8.629$; $P=.004$; $\eta^2=0.108$).

With regard to the depression measure, the results of ANCOVA with repeated measures yielded a significant main effect of time ($F=3.892$; $P=.05$; $\eta^2=0.052$), while controlling for the past 3-month therapist visit. The results indicated that the 2 groups showed a significant reduction in depressive symptoms from baseline to after the intervention. In contrast, the ANCOVA with repeated measures revealed no significant main effect of group and no significant interaction effect of time with group. The resulting MDI classification for both groups after the intervention was still considered within the no or doubtful range for depression severity. Similarly, the 3-month therapist visit was a significant covariate for depression ($F=7.007$; $P=.01$; $\eta^2=0.090$)."

17a) For each primary and secondary outcome, results for each group, and the estimated effect size and its precision (such as 95% confidence interval)

Does your paper address CONSORT subitem 17a? *

Copy and paste relevant sections from the manuscript (include quotes in quotation marks "like this" to indicate direct quotes from your manuscript), or elaborate on this item by providing additional information not in the ms, or briefly explain why the item is not applicable/relevant for your study

"For the anxiety measure, while controlling for the past 3-month therapist visit, the results of ANCOVA with repeated measures indicated no significant main effect of time and group and no significant interaction between time and group. However, the main effect of time was close to a significant level ($F=3.485$; $P=.07$; $\eta 2=0.047$), indicating that the 2 groups had a marginally significant decrease in anxiety over time. The resulting GAD-7 classification for both groups after the intervention was still considered within the mild range for anxiety severity. The results also showed that the past 3-month therapist visit was a significant covariate of anxiety ($F=8.629$; $P=.004$; $\eta 2=0.108$).

With regard to the depression measure, the results of ANCOVA with repeated measures yielded a significant main effect of time ($F=3.892$; $P=.05$; $\eta 2=0.052$), while controlling for the past 3-month therapist visit. The results indicated that the 2 groups showed a significant reduction in depressive symptoms from baseline to after the intervention. In contrast, the ANCOVA with repeated measures revealed no significant main effect of group and no significant interaction effect of time with group. The resulting MDI classification for both groups after the intervention was still considered within the no or doubtful range for depression severity. Similarly, the 3-month therapist visit was a significant covariate for depression ($F=7.007$; $P=.01$; $\eta 2=0.090$)."

17a-i) Presentation of process outcomes such as metrics of use and intensity of use

In addition to primary/secondary (clinical) outcomes, the presentation of process outcomes such as metrics of use and intensity of use (dose, exposure) and their operational definitions is critical. This does not only refer to metrics of attrition (13-b) (often a binary variable), but also to more continuous exposure metrics such as “average session length”. These must be accompanied by a technical description how a metric like a “session” is defined (e.g., timeout after idle time) [1] (report under item 6a).

	1	2	3	4	5	
subitem not at all important	(●)	( )	( )	( )	( )	essential

Clear selection

Does your paper address subitem 17a-i?

Copy and paste relevant sections from the manuscript (include quotes in quotation marks "like this" to indicate direct quotes from your manuscript), or elaborate on this item by providing additional information not in the ms, or briefly explain why the item is not applicable/relevant for your study

Not applicable to this study

17b) For binary outcomes, presentation of both absolute and relative effect sizes is recommended

Does your paper address CONSORT subitem 17b? *

Copy and paste relevant sections from the manuscript (include quotes in quotation marks "like this" to indicate direct quotes from your manuscript), or elaborate on this item by providing additional information not in the ms, or briefly explain why the item is not applicable/relevant for your study

Not applicable to this study

18) Results of any other analyses performed, including subgroup analyses and adjusted analyses, distinguishing pre-specified from exploratory

Does your paper address CONSORT subitem 18? *

Copy and paste relevant sections from the manuscript (include quotes in quotation marks "like this" to indicate direct quotes from your manuscript), or elaborate on this item by providing additional information not in the ms, or briefly explain why the item is not applicable/relevant for your study

Not applicable to this study

18-i) Subgroup analysis of comparing only users

A subgroup analysis of comparing only users is not uncommon in ehealth trials, but if done, it must be stressed that this is a self-selected sample and no longer an unbiased sample from a randomized trial (see 16-iii).

	1	2	3	4	5	
subitem not at all important	◉	○	○	○	○	essential

Clear selection

Does your paper address subitem 18-i?

Copy and paste relevant sections from the manuscript (include quotes in quotation marks "like this" to indicate direct quotes from your manuscript), or elaborate on this item by providing additional information not in the ms, or briefly explain why the item is not applicable/relevant for your study

Not applicable to this study

19) All important harms or unintended effects in each group

(for specific guidance see CONSORT for harms)

Does your paper address CONSORT subitem 19? *

Copy and paste relevant sections from the manuscript (include quotes in quotation marks "like this" to indicate direct quotes from your manuscript), or elaborate on this item by providing additional information not in the ms, or briefly explain why the item is not applicable/relevant for your study

Not mentioned in the manuscript

19-i) Include privacy breaches, technical problems

Include privacy breaches, technical problems. This does not only include physical “harm” to participants, but also incidents such as perceived or real privacy breaches [1], technical problems, and other unexpected/unintended incidents. “Unintended effects” also includes unintended positive effects [2].

	1	2	3	4	5	
subitem not at all important	(●)	( )	( )	( )	( )	essential

Clear selection

Does your paper address subitem 19-i?

Copy and paste relevant sections from the manuscript (include quotes in quotation marks "like this" to indicate direct quotes from your manuscript), or elaborate on this item by providing additional information not in the ms, or briefly explain why the item is not applicable/relevant for your study

Not applicable to this study

19-ii) Include qualitative feedback from participants or observations from staff/researchers

Include qualitative feedback from participants or observations from staff/researchers, if available, on strengths and shortcomings of the application, especially if they point to unintended/unexpected effects or uses. This includes (if available) reasons for why people did or did not use the application as intended by the developers.

	1	2	3	4	5	
subitem not at all important	(●)	( )	( )	( )	( )	essential

Clear selection

Does your paper address subitem 19-ii?

Copy and paste relevant sections from the manuscript (include quotes in quotation marks "like this" to indicate direct quotes from your manuscript), or elaborate on this item by providing additional information not in the ms, or briefly explain why the item is not applicable/relevant for your study

Not applicable to this study

DISCUSSION

22) Interpretation consistent with results, balancing benefits and harms, and considering other relevant evidence

NPT: In addition, take into account the choice of the comparator, lack of or partial blinding, and unequal expertise of care providers or centers in each group

22-i) Restate study questions and summarize the answers suggested by the data, starting with primary outcomes and process outcomes (use)

Restate study questions and summarize the answers suggested by the data, starting with primary outcomes and process outcomes (use).

	1	2	3	4	5	
subitem not at all important	○	○	○	○	◉	essential

Clear selection

Does your paper address subitem 22-i? *

Copy and paste relevant sections from the manuscript (include quotes in quotation marks "like this" to indicate direct quotes from your manuscript), or elaborate on this item by providing additional information not in the ms, or briefly explain why the item is not applicable/relevant for your study

"This study aims to examine the effects of a web-based aerobic resistance exercise (WeActive) intervention and web-based yoga mindfulness (WeMindful) intervention on college students’ anxiety and depression levels. Both the 8-week interventions were hypothesized to have positive effects on anxiety and depression, with greater positive effects predicted in the WeActive group. As expected, the participants in the 2 groups experienced a significant decrease in depression scores over the 8-week period. Although not statistically significant, the participants experienced a decrease in anxiety scores as well. Contrary to our second hypothesis, there were no significant interactions between group and time, indicating that the WeActive group did not experience greater improvements in mental health scores than the WeMindful group over time"

22-ii) Highlight unanswered new questions, suggest future research

Highlight unanswered new questions, suggest future research.

	1	2	3	4	5	
subitem not at all important	○	○	○	○	◉	essential

Clear selection

Does your paper address subitem 22-ii?

Copy and paste relevant sections from the manuscript (include quotes in quotation marks "like this" to indicate direct quotes from your manuscript), or elaborate on this item by providing additional information not in the ms, or briefly explain why the item is not applicable/relevant for your study

"future research could benefit from exploring single modality interventions, such as aerobic exercise compared with resistance training or yoga compared with meditation. This study suggests that both the combination of aerobic resistance training and the combination of yoga mindfulness exercises are associated with a reduction in college student depression scores over an 8-week period. As the findings from our study indicate the potential efficacy of WeActive and WeMindful interventions in improving student mental health measures, this study design could be a useful resource for colleges and mental health treatment providers."

20) Trial limitations, addressing sources of potential bias, imprecision, and, if relevant, multiplicity of analyses

20-i) Typical limitations in ehealth trials

Typical limitations in ehealth trials: Participants in ehealth trials are rarely blinded. Ehealth trials often look at a multiplicity of outcomes, increasing risk for a Type I error. Discuss biases due to non-use of the intervention/usability issues, biases through informed consent procedures, unexpected events.

	1	2	3	4	5	
subitem not at all important	○	○	○	○	◉	essential

Clear selection

Does your paper address subitem 20-i? *

Copy and paste relevant sections from the manuscript (include quotes in quotation marks "like this" to indicate direct quotes from your manuscript), or elaborate on this item by providing additional information not in the ms, or briefly explain why the item is not applicable/relevant for your study

"This study has several limitations...." - complete limitations section provided in the manuscript

21) Generalisability (external validity, applicability) of the trial findings

NPT: External validity of the trial findings according to the intervention, comparators, patients, and care providers or centers involved in the trial

21-i) Generalizability to other populations

Generalizability to other populations: In particular, discuss generalizability to a general Internet population, outside of a RCT setting, and general patient population, including applicability of the study results for other organizations

	1	2	3	4	5	
subitem not at all important	○	○	○	○	◉	essential

Clear selection

Does your paper address subitem 21-i?

Copy and paste relevant sections from the manuscript (include quotes in quotation marks "like this" to indicate direct quotes from your manuscript), or elaborate on this item by providing additional information not in the ms, or briefly explain why the item is not applicable/relevant for your study

Limitations section includes some discussion of generalizability of results

21-ii) Discuss if there were elements in the RCT that would be different in a routine application setting

Discuss if there were elements in the RCT that would be different in a routine application setting (e.g., prompts/reminders, more human involvement, training sessions or other co-interventions) and what impact the omission of these elements could have on use, adoption, or outcomes if the intervention is applied outside of a RCT setting.

	1	2	3	4	5	
subitem not at all important	(•)	( )	( )	( )	( )	essential

Clear selection

Does your paper address subitem 21-ii?

Copy and paste relevant sections from the manuscript (include quotes in quotation marks "like this" to indicate direct quotes from your manuscript), or elaborate on this item by providing additional information not in the ms, or briefly explain why the item is not applicable/relevant for your study

Not included in this manuscript

OTHER INFORMATION

23) Registration number and name of trial registry

Does your paper address CONSORT subitem 23? *

Copy and paste relevant sections from the manuscript (include quotes in quotation marks "like this" to indicate direct quotes from your manuscript), or elaborate on this item by providing additional information not in the ms, or briefly explain why the item is not applicable/relevant for your study

approved by the University Institutional Review Board-Health Sciences and Behavioral Sciences (IRB-HSBS) (HUM00189120)

24) Where the full trial protocol can be accessed, if available

Does your paper address CONSORT subitem 24? *

Cite a Multimedia Appendix, other reference, or copy and paste relevant sections from the manuscript (include quotes in quotation marks "like this" to indicate direct quotes from your manuscript), or elaborate on this item by providing additional information not in the ms, or briefly explain why the item is not applicable/relevant for your study

Accessible by contacting the researchers

25) Sources of funding and other support (such as supply of drugs), role of funders

Does your paper address CONSORT subitem 25? *

Copy and paste relevant sections from the manuscript (include quotes in quotation marks "like this" to indicate direct quotes from your manuscript), or elaborate on this item by providing additional information not in the ms, or briefly explain why the item is not applicable/relevant for your study

Addressed in additional documentation submitted to publisher

X27) Conflicts of Interest (not a CONSORT item)

X27-i) State the relation of the study team towards the system being evaluated

In addition to the usual declaration of interests (financial or otherwise), also state the relation of the study team towards the system being evaluated, i.e., state if the authors/evaluators are distinct from or identical with the developers/sponsors of the intervention.

	1	2	3	4	5	
subitem not at all important	○	○	○	○	◉	essential

Clear selection

Does your paper address subitem X27-i?

Copy and paste relevant sections from the manuscript (include quotes in quotation marks "like this" to indicate direct quotes from your manuscript), or elaborate on this item by providing additional information not in the ms, or briefly explain why the item is not applicable/relevant for your study

There were no conflicts of interest

About the CONSORT EHEALTH checklist

As a result of using this checklist, did you make changes in your manuscript? *

- ○ yes, major changes
- ○ yes, minor changes
- ◉ no

What were the most important changes you made as a result of using this checklist?

Your answer

How much time did you spend on going through the checklist INCLUDING making changes in your manuscript *

Spent 30+ minutes completing the checklist

As a result of using this checklist, do you think your manuscript has improved? *

- ( ) yes
- ( ) no
- (•) Other: Knowing what to look for in future manuscripts has improved

Would you like to become involved in the CONSORT EHEALTH group?

This would involve for example becoming involved in participating in a workshop and writing an "Explanation and Elaboration" document

- ( ) yes
- (•) no
- ( ) Other:

Clear selection

Any other comments or questions on CONSORT EHEALTH

Your answer

STOP - Save this form as PDF before you click submit

To generate a record that you filled in this form, we recommend to generate a PDF of this page (on a Mac, simply select "print" and then select "print as PDF") before you submit it.

When you submit your (revised) paper to JMIR, please upload the PDF as supplementary file.

Don't worry if some text in the textboxes is cut off, as we still have the complete information in our database. Thank you!

Final step: Click submit !

Click submit so we have your answers in our database!

Submit

Clear form

Never submit passwords through Google Forms.

This content is neither created nor endorsed by Google. Report Abuse - Terms of Service - Privacy Policy

Google Forms
